# Exosomes from liver progenitor cells carrying JAG1 activate notch signaling to promote liver regeneration in PVL rats

**DOI:** 10.1038/s41419-025-07925-1

**Published:** 2025-08-12

**Authors:** Mingqi Liu, Yan Zhu, Zhishuai Li, Yong Yu, Duoxiang Wang, Yue Wang, Xiaoqing Jiang, Bin Li

**Affiliations:** 1 Department I of Biliary Tract, The Third Affiliated Hospital of Naval Medical University, Eastern Hepatobiliary Surgery Hospital, Shanghai, China; 2 Department of Biliary-Pancreatic Surgery, Sun Yat-sen Memorial Hospital, Sun Yat-sen University, Guangzhou, China; 3 Department of Pathology, The First Affiliated Hospital of Naval Medical University, Changhai Hospital, Shanghai, China; 4 Department of Pathology, No. 905 Hospital of People’s Liberation Army Navy of Naval Medical University, Shanghai, China; 5 Waite Research Institute, School of Agriculture, Food and Wine, The University of Adelaide, Waite campus, Adelaide, SA Australia; 6 Stem Cell and Regeneration Medicine Institute, Research Center of Translational Medicine, Naval Medical University, Shanghai, China; 7 Department of Stem Cell Engineering, Shanghai Key Laboratory of Cell Engineering, Naval Medical University, Shanghai, China

**Keywords:** Liver diseases, HIPPO signalling, Regeneration, ESCRT, Transport carrier

## Abstract

Studies have shown that extracellular vesicles play a crucial role in maintaining homeostasis in healthy individuals and influencing disease pathology in patients. However, the mechanisms by which exosomes facilitate liver regeneration following portal vein ligation (PVL) remain unclear. Our previous research highlighted the critical role of the Notch signaling pathway in liver regeneration after PVL. Nevertheless, the involvement of liver-derived exosomes in actively promoting regeneration through the YAP-Notch signaling pathway and their role in cell proliferation have not been fully explored. In this study, we demonstrate that exosomes from hypertrophic liver tissue can activate the YAP-Notch signaling pathway both in vitro and in vivo, thereby promoting liver regeneration after PVL. Specifically, we show that these exosomes carry JAG1, which activates Notch signaling in recipient cells, a process that is inhibited by JAG1 antibodies. Co-immunoprecipitation and mass spectrometry (Co-IP-MS) identified JAG1 interactors, confirming that ALG-2 plays a critical role in linking SEC31A and Alix to facilitate the intracellular transport and sorting of JAG1 onto exosomes. Additionally, we reveal that YAP induces hepatocyte reprogramming into Sox9^+^ liver progenitor cells (LPCs) and promotes the release of JAG1^+^ exosomes via the SEC31A/ALG-2/Alix axis, thereby activating Notch signaling in neighboring cells and enhancing liver regeneration. These findings suggest that YAP^+^Sox9^+^ LPCs serve as a key source of JAG1^+^ exosomes, playing a vital role in liver regeneration following PVL.

## Introduction

The liver, often regarded as a chemical factory of the body, plays a crucial role in nutrient metabolism, detoxification, regulation of coagulation factors, and bile synthesis [1]. When drugs or hepatotoxins cause significant damage, its regenerative capacity can be overwhelmed, leading to acute or chronic liver failure [2]. Insufficient liver remnant volume after surgery is the main factor contributing to liver failure and increased risk of complications [3]. In 1990, Makuuchi introduced portal vein embolization (PVE), to effectively increase liver volume in clinical practice [4]. PVE induces atrophy in the embolized liver lobe while promoting compensatory hypertrophy in the non-embolized lobe, reducing mortality in extensive liver resections and improving the resectability of previously inoperable large liver cancers [5, 6]. However, the mechanisms through which exosomes facilitate liver regeneration after portal vein ligation (PVL) /PVE remain unclear.

Studies have shown that extracellular vesicles (EVs) play a crucial role in maintaining homeostasis in healthy individuals and influence disease pathology in patients [7]. In liver diseases, exosomes from different cell types and disease models play distinct roles in various biological processes [8, 9]. Exosomes derived from mesenchymal stem cells (MSCs) have been shown to promote regeneration in acute and chronic liver diseases, as well as after hepatectomy, by regulating signaling molecules essential for hepatocyte proliferation [10, 11]. Exosomes derived from hepatocytes promote a dose-dependent increase in hepatocyte proliferation, whereas exosomes from Kupffer cells and LSECs do not induce this effect [12]. Both normal and damaged liver EVs significantly accelerate the recovery of liver tissue from CCl_4_-induced hepatic necrosis [13]. These studies have demonstrated that various types of EVs are crucial for liver regeneration and repair.

Our previous research demonstrated that the Notch signaling pathway may play a critical role in liver regeneration after PVL [14]. After partial hepatectomy (PH), the Notch receptor, ligand JAG1, and other related genes or proteins are upregulated in the early stages [15, 16]. Yes-associated protein (YAP) is crucial for liver size regulation and regeneration [17, 18]. Notch receptor and ligand JAG1 are direct target genes of YAP-TEAD [19–21]. YAP activation in hepatocytes induces the transformation of mature hepatocytes into Sox9^+^HNF4α^+^ liver progenitor cells (LPCs), which can subsequently redifferentiate into hepatocyte and cholangiocyte lineages [22–24]. Exosomes from TGF-β1-pretreated MSCs enhance the alleviation of oxidative stress, apoptosis, and inflammation in biliary ischemia-reperfusion injury repair via the Jagged1/Notch/Sox9 pathway [25]. Exosomes from HIF-1α-overexpressing MSCs activate Notch signaling and induce angiogenesis in endothelial cells, partly by enhancing JAG1 packaging [26]. Exosomes from a stiff matrix show increased JAG1 protein expression, promoting tumor proliferation and invasion via Notch pathway activation [27]. Whether the YAP-Notch pathway is activated by exosomes after PVL and whether YAP activity affects JAG1 sorting onto exosomes remains unknown.

In this study, we demonstrated that after PVL, YAP-Notch activation was confirmed, along with the finding that liver-derived exosomes activate this pathway and carry JAG1, thereby promoting liver regeneration through Notch activation. ALG-2 was identified and confirmed as a key protein linking SEC31A and Alix, facilitating the sorting of JAG1 onto exosomes. YAP induced the reprogramming of hepatocytes into Sox9^+^ LPCs and promoted the release of JAG1^+^ exosomes via the SEC31A/ALG-2/Alix axis, thereby activating Notch signaling to enhance liver regeneration.

## Material and methods

### Animals

Male Sprague Dawley (SD) rats weighing 200–250 g were obtained from Shanghai Bikai Keyi Biotechnology Co., Ltd. and housed at the Animal Center of Naval Medical University under specific pathogen-free conditions. Care, breeding, and experimental procedures adhered to the guidelines of the Ethics Committee. The breeding room was maintained at a temperature of 23 °C, 50% relative humidity, and a 12 h light-dark cycle. Before conducting the animal experiments, sample size estimation was performed based on preliminary data and published literature. A minimum of three biological replicates per group (*n* = 3) was chosen to ensure statistical reliability and experimental reproducibility.

### PVL surgery and specimen collection

**Fig. 1:**
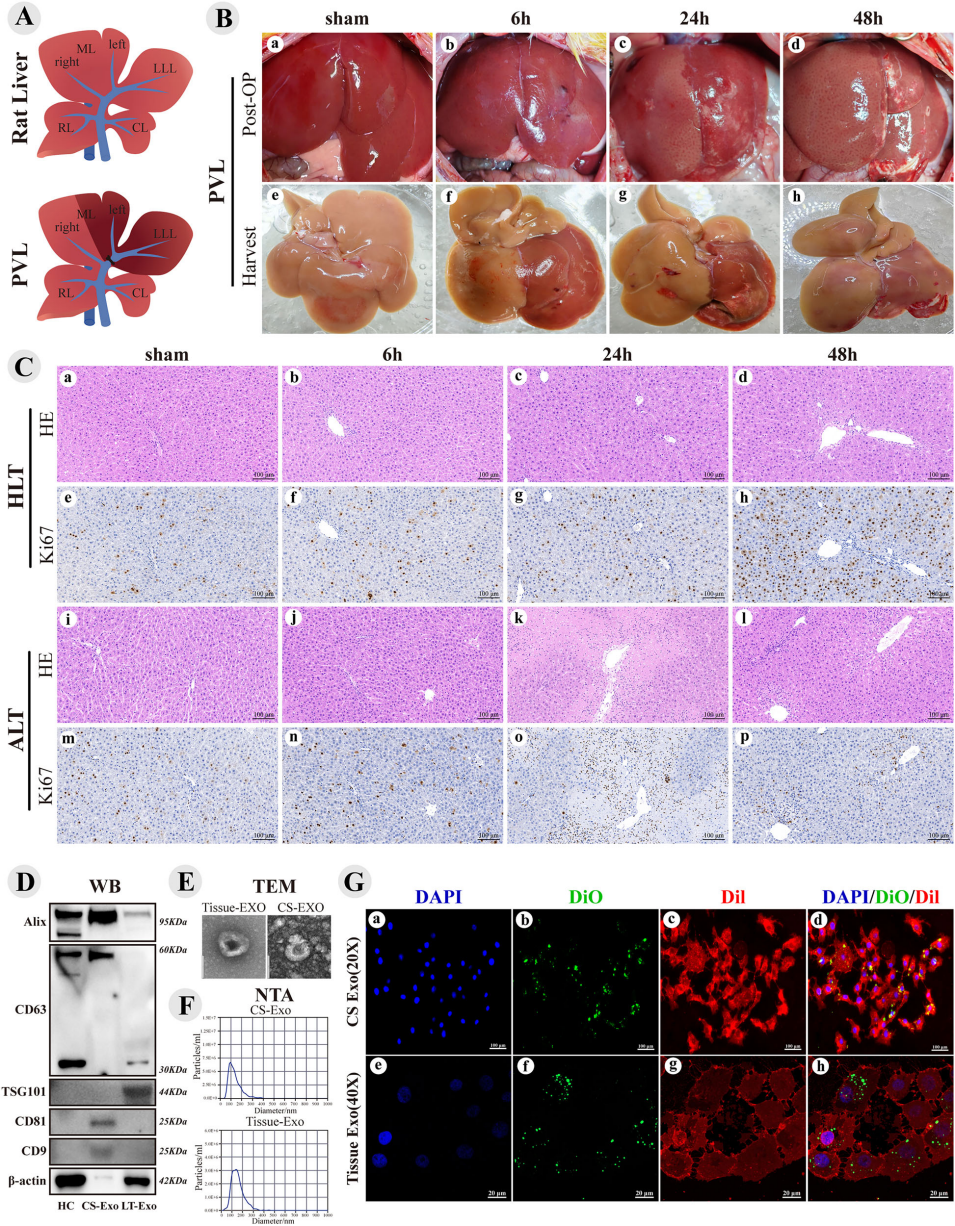
PVL surgery and assessment, Exosomes isolation and identification. **A** Normal rat liver and PVL surgery. **B** **a–d** show liver images after surgery. **e–h** display liver specimens harvested after perfusion. **C** Hypertrophic Liver Tissue (HLT), Atrophic Liver Tissue (ALT). H&E and Ki67 were used to characterize liver repair following surgery. **D** Hepatocyte (HC, BRL-3A), BRL-3A cell line supernatant exosomes (CS-Exo), Liver tissue exosomes (LT-Exo). Immunoblotting of exosome markers. **E** TEM images of exosomes reveal oval-shaped membrane structures resembling red blood cells. **F** The NTA results for exosomes show a unimodal curve, with most diameters between 50 and 200 nm. **G** Green indicates DiO-stained exosomes, red represents the BRL-3A cell membrane stained with Dil, and blue shows DAPI-stained nuclei. **a–d** illustrate the uptake of CS-Exo (Exosomes from the supernatant of BRL-3A cells) using a standard fluorescence microscope, while figures **e–h** depict the uptake of Tissue-Exo (liver tissue exosomes) captured with a confocal fluorescence microscope.

After intraperitoneal injection of pentobarbital, intra-abdominal bleeding, injuries, and anatomical variations were assessed. The porta hepatis were dissected to identify Glisson’s capsules in the various liver lobes. The left lateral lobe and branches of the middle-left portal vein were isolated and ligated, revealing clear ischemic lines. No active bleeding was observed, and the incision was sutured (Fig. 1A). The sham group underwent a procedure that involved only laparotomy and exploration of the porta hepatis and liver, followed by immediate harvesting of the liver tissue to eliminate experimental errors caused by anesthesia and surgical effects. Other liver tissue specimens were collected, 6, 24, and 48 h after surgery (Fig. 1B). Phosphate-buffered saline (PBS) was perfused through the left ventricle and the portal vein. Once the liver turned clay-colored, it was carefully extracted and placed in a 10 cm Petri dish with cold PBS on ice. Blood stains were removed, and a portion of the tissue was excised and preserved in 4% paraformaldehyde for 24 h before being sent for pathological embedding for immunohistochemistry (IHC) and immunofluorescence (IF) procedures. Tissue of mung bean size was divided for Western blot (WB) analysis and reverse transcription quantitative polymerase chain reaction (RT-qPCR). The remaining tissue was cut into fragments, placed in a cryovial, and transferred to a liquid nitrogen tank for storage at −80 °C, enabling subsequent isolation of tissue exosomes.

### Cell culture and supernatant collection

BRL-3A cells were obtained from the Cell Bank of the Chinese Academy of Sciences. The complete culture medium contained 450 mL DMEM (BasalMedia, L110KJ), 50 mL FBS (Servicebio, G8002), and 5 mL penicillin/streptomycin (Gibco, 15140-122). The supernatant for exosomes isolation was cultured in exosomes-free serum. When the cells reached 80–90% confluence, the supernatant was harvested, stored at −80 °C, and thawed at 4 °C before exosomes isolation.

### Cell supernatant and tissue exosomes isolation

After thawing, the cell supernatant was centrifuged at 4 °C, 2000 g for 20 min to remove debris. The supernatant was centrifuged again at 4 °C, 16,500 g for 45 min. After filtration through a 0.22 μm filter, it underwent ultracentrifugation at 4 °C, 100,000 g for 2 h. The sediment was resuspended in PBS and subjected to ultracentrifugation at 4 °C, 100,000 g for 2 h. The isolated exosomes were resuspended in PBS, aliquoted into 1.5 mL EP tubes, and stored at −80 °C. Exosomes from liver tissue were isolated using enzymatic hydrolysis and ultracentrifugation [28], employing Medium 1640 (Sigma-Aldrich, R7388-500ML), Collagenase D (Roche, 11088858001), and DNase I (Roche, 11284932001). Liver specimens were dissected into 2 mm³ cubic fragments and enzymatically digested with Collagenase D (1 mg/mL) and DNase I (0.1 mg/mL) in 1640 medium at 37 °C for 30 min. The resultant homogenate was filtered through a 70 μm nylon mesh filter to remove undigested tissue debris. After primary centrifugation (300 × g, 10 min, 4 °C), the supernatant was carefully decanted into a 50 mL conical tube with subsequent steps performed at room temperature. All subsequent centrifugation steps adhered to this protocol for cellular supernatant processing.

### RNA extraction and RT-qPCR

RNA extraction from tissues and cells was performed using TRIzol reagent (Takara, 9109). Tissue samples were ground into a powder with liquid nitrogen, and cells in a 6-well plate were washed with PBS before adding TRIzol. The subsequent steps were performed according to the manufacturer’s instructions. The RNA pellet was dried and dissolved in RNase-free water, and its concentration was measured using a NanoDrop spectrophotometer. One microgram of RNA was reverse-transcribed into cDNA using HiScript III RT SuperMix for qPCR (+gDNA wiper) (Vazyme, R323). RT-qPCR was performed using the Taq Pro Universal SYBR qPCR Master Mix (Vazyme, Q712) according to the manufacturer’s protocol. β-actin served as the internal reference control for mRNA, and primer sequences are provided in the supplementary data (Primer sequences).

### Cell proliferation assays

Cell proliferation assays were performed using the Enhanced Cell Counting Kit-8 (CCK8) (Beyotime, C0043) and BeyoClick™ EdU Cell Proliferation Kit with Alexa Fluor 555 (EdU) (Beyotime, C0075S) according to the manufacturer’s instructions. For the CCK8 assay, cells were seeded in a 96-well plate with five replicates per group and incubated for 24 h. Different concentrations of drugs or exosomes filtered through a 0.22 μm filter were added, and the cells were cultured for an additional 24 and 48 h. After changing the medium, CCK8 solution was added, and the cells were incubated for an additional 2 h. The absorbance at 450 nm was measured using a Microplate Reader. The EdU assay was performed in accordance with the manufacturer’s guidelines. Fluorescence images of Hoechst 33342 and Azide 555 were captured at 48 h, and ImageJ software was used to calculate the ratio of EdU-positive to nuclear stain-positive cells.

### Western Blot

Protein expression levels, including exosome marker proteins, were assessed by WB analysis. Liver tissue, cells, and exosome proteins were separated on a Precast Protein Plus Gel (4–20%, 15 wells, Yeasen, 36270ES10) and transferred to a polyvinylidene fluoride (PVDF) membrane (Millipore, IPVH00010) at 400 mA. The membrane was blocked with 5% defatted milk for 1 h, followed by overnight incubation with the primary antibodies at 4 °C. After three washes with TBST, the membranes were incubated with secondary antibody for 2 h at room temperature. Immunobands were visualized using the Immobilon Western Chemilum HRP substrate (ShareBio, SB-WB004). Details of the primary and secondary antibodies used are provided in the supplementary data (Antibodies).

### Transmission electron microscope (TEM)

TEM (HITACHI, Japan) was used to visualize the morphology of exosomes. 10 μL of the exosome sample was placed on a copper grid for 1 min to allow for precipitation. Excess liquid was removed with filter paper, and 10 μL of uranyl acetate was applied for another minute, followed by the removal of the excess solution. The grid was air-dried at room temperature for a few minutes before imaging.

### Nanoparticle Tracking Analysis (NTA)

Particle sizes of the exosome samples were determined using NTA (Particle Metrix, Germany). The sample pool was initially cleaned with ddH₂O, and the instrument was calibrated using polystyrene microspheres (100 nm, 1:250,000 dilution). The instrument was then washed again with PBS. The exosome sample was diluted with 1×PBS, with sample A diluted 50,000 times, and sample B diluted 30,000 times. Both samples were tested, and the dilution factor was adjusted based on their concentration. Finally, samples were photographed for further observation.

### Exosome Fluorescent Uptake Experiment

BRL-3A cells were seeded on slides in 24-well plate and cultured for 24 h. DiO (Beyotime, C1993S) was added to the exosomes at 1:100 dilution and incubated at 37 °C in the dark for 30 min. Exosomes were washed with PBS by centrifugation at 10,000 g at 4 °C for 15 min, repeated four times, and resuspended in PBS. The DiO-stained exosomes were incubated with JAG1 primary antibody at a 1:100 dilution overnight at 4 °C. After washing, the appropriate secondary antibody was added to the JAG1-exosomes and incubated at 4 °C for 4 h, followed by another wash step. The medium was replaced with exosome-free complete serum medium, and labeled exosomes were added for an additional 12 h. After two washes with PBS, 300 μL of Dil (Beyotime, C1991S) was added (noting that JAG1-exosomes did not stain the membrane) and the cells were incubated at 37 °C for 20 min in the dark. The cells were washed thrice with PBS, followed by Hoechst staining of the nuclei. After a 10 min incubation at 37 °C, the cells were washed three times with PBS and fixed with 200 μL of 4% PFA at room temperature for 15 min. After three washes with PBS, the slides were removed, sealed with PBS, and observed and photographed using a fluorescence or confocal microscope.

### Lentivirus transfection

Lentiviruses were purchased from Hanheng Biotechnology Co., Ltd. (Shanghai). BRL-3A cells were counted and seeded into a 6-well plate at a density of 0.8 × 10^5^ cells per well and cultured for 24 h. The next day, the cells were infected at a multiplicity of infection (MOI) of 30. After 24 h, fresh virus-free complete medium was added to cells. At 48 h post-infection, the cells were transferred to 10 cm dishes and allowed to adhere for 6 h before adding puromycin (1.5 μg/mL) to screen for polyclonal stable transfectants. After drug screening, the cells were passaged, cryopreserved, and used in the experiments one week later.

### Detection of Cells Drug IC50

BRL-3A cells were counted and seeded in a 96-well plate at a density of 3000 cells per well, followed by 24 h of culture. Verteporfin (Millipore, SML0534) was prepared as a stock solution at 20 μM according to the manufacturer’s instructions. Nine drug concentration gradients were established, 10, 5, 2.5, 1.25, 0.625, 0.3125, 0.15625, and 0.078125 μM. Each concentration was tested in five replicate wells with the respective drugs added for 48 h. The culture medium was then replaced with 100 μL of medium containing 10% CCK8 reagent and incubated for an additional 2 h. Absorbance at 450 nm was measured using a Microplate Reader. The IC50 was calculated using the following equation: Cell viability = (OD_Drug_ – OD_Blank_) / (OD_Ctrl_ – OD_Blank_) × 100% and Cell inhibition = 1 - Cell viability.

### Co-immunoprecipitation (Co-IP)

The Co-IP kit used was the Immunoprecipitation Kit with Protein A + G Magnetic Beads (Beyotime, P2179M). A cell lysis solution was prepared by mixing Lysis Buffer and Protease Inhibitor Cocktail at a 100:1 ratio and kept on ice. A 100 mg sample of frozen liver tissue was retrieved from the −80 °C freezer, immersed in liquid nitrogen, and ground into a fine powder using a handheld homogenizer. The lysis solution was added at 20 mg per 200 μL, and the mixture was vortexed until it was fully lysed. The mixture was then incubated on ice for 60 min and vortexed every 10 min. The sample was centrifuged at 10,000 g and 4 °C for 10 min, the supernatant was collected for analysis, and the protein concentration was measured. 20 μL of protein A/G magnetic beads were prepared for both immunoprecipitation (IP) and immunoglobulin G (IgG) samples. Each group received 0.5 mL of 1× TBS, mixed by inverting the tube ten times. The tube was placed on a magnetic stand for 10 s to collect the beads and the supernatant was removed. This washing step was repeated thrice. Next, 500 μL of TBS diluted with 1 μg of experimental-grade antibody was added to the IP beads, and the same amount of IgG antibody was added to the IgG beads. The mixtures were then incubated for 1 h at room temperature on a rotating rack. After incubation, 500 μL of TBS was added to resuspend the beads for washing. The tubes were then placed on a magnetic stand to aspirate the supernatant, and the washing process was repeated three times. Finally, 20 μL TBS was added to resuspend the magnetic beads. Then, the prepared tissue lysate (0.5 mL) was added to the corresponding magnetic beads for both IP and IgG samples. The setup was incubated overnight at 4 °C on a rotating rack. Additionally, 0.1 mL of the Input sample was stored in a −80 °C freezer as a backup. After overnight incubation, 10 μL of protein A/G magnetic beads was added, and the samples were incubated at room temperature for 1 h. 0.5 mL of Lysis Buffer containing protease and phosphatase inhibitors was added to the IP and IgG samples. The magnetic beads were thoroughly mixed and placed on a magnet for 10 s. The supernatant was aspirated, and the process was repeated three times. The cleanliness of the washing liquid was assessed by measuring the optical density at 280 nm (OD280); if the OD280 exceeded 0.05, additional wash cycles were performed. Afterward, 100 μL of SDS-PAGE Sample Loading Buffer (1×) was added to every 20 μL of magnetic beads, and the protein was eluted by boiling the mixture for 5 min. The Input sample was retrieved from the −80 °C freezer and thawed on ice. Five times the volume of SDS-PAGE Sample Loading Buffer was added to achieve a final concentration of 1×, and the mixture was boiled at 95 °C for 5 min. The loading amount for the Input was 40 μg per well, whereas both IP and IgG samples were loaded at 10 μL per well of the eluted protein stock solution. Further steps followed the WB protocol.

### SDS-PAGE silver staining

The Fast Silver Stain Kit (Beyotime, P0017S) was used for silver staining. After electrophoresis, the gel was removed and treated with 100 mL of silver stain fixative and shaken at 60–70 rpm for 20 min (this can be extended to 40 min or overnight to reduce background staining). The fixative was discarded and the gel was washed with 100 mL of 30% ethanol for 10 min. Subsequently, the gel was rinsed with 200 mL of ddH₂O for 10 min. Next, 100 mL of silver stain sensitizing solution was added and shaken for 2 min, followed by another ddH₂O wash for 1 min. Then, 100 mL of silver solution was added and shaken for 10 min, after which it was discarded. The gel was washed with water for 1.5 min, then treated with 100 mL of silver stain solution for 3–10 min until clear protein bands appeared. Finally, the silver stain solution was discarded and 100 mL of the termination solution was added and shaken for 10 min. After another wash with 100 mL of ddH₂O for 2–5 min, photographs were taken under bright light for storage.

### H&E staining

To prepare paraffin-fixed specimens, the sections were sliced and immersed in environmentally friendly dewaxing liquid I for 20 min, followed by immersion in liquid II for another 20 min. The slices were then placed in absolute ethanol I and II for 5 min each, transferred to a 75% alcohol solution for 5 min, and rinsed with tap water. Next, the slices were treated with a constant stain pretreatment solution for 1 min. Dewaxed and dehydrated sections were submerged in hematoxylin dye for 3–5 min, washed with tap water, and treated with differentiation solution. After rinsing, the sections were placed in a bluing solution until a blue hue developed and then rinsed with running water. For eosin staining, the sections were dehydrated in 95% alcohol for 1 min and then placed in eosin staining solution for 15 s. They were sequentially treated with absolute ethanol I, II, III, n-butanol I, II, and xylene I, II, each for 2 min, before sealing with rubber. Finally, the sections were observed under a fluorescence microscope and photographs were taken for storage if they met the required standards.

### Immunohistochemistry (IHC)

Tissue sections, typically 4 μm thick, were cut using a microtome. They were immersed in environment-friendly dewaxing liquids I, II, and III for 10 min each, followed by immersion in absolute ethanols I, II, and III for 5 min each, and then washed with distilled water. After high-pressure boiling in citric acid repair solution (pH 6.0), the sections were incubated for 3 min under cover, rinsed with tap water, cooled, and washed three times in PBS (pH 7.4) for 5 min each. The sections were incubated in 3% hydrogen peroxide in the dark for 25 min at room temperature, followed by three washes in PBS (pH 7.4) for 5 min each. Tissue outlines were marked, and 3% BSA serum was applied for blocking using species-specific serum as necessary. The sections were blocked for 30 min at room temperature to ensure coverage. After blotting the blocking solution, the primary antibody working solution was added. The sections were incubated overnight at 4 °C in a humidity chamber, washed three times in PBS (pH 7.4) for 5 min each, and treated with HRP-conjugated secondary antibody for 50 min at room temperature. Fresh DAB staining solution was added, and the sections were monitored until a brownish color developed, after which they were rinsed with tap water. The sections were counterstained with hematoxylin for 3 min, washed with tap water, and treated with differentiation solution for a few seconds. They were then rinsed and treated with bluing solution, followed by thorough washing with running water. The stained sections were dehydrated by sequential immersion in 75% ethanol, 85% ethanol, absolute ethanol I, II, n-butanol, and xylene I, each for 5 min each. After air drying, the samples were mounted and observed under a fluorescence microscope, and photographs were taken if the staining was satisfactory.

### Immunofluorescence (IF)

Tissue sections, typically 4 μm thick, were cut using a microtome. They were immersed in environmentally friendly dewaxing liquids I, II, and III for 10 min each, followed by immersion in absolute ethanol I, II, and III for 5 min each, and rinsing with distilled water. After high pressure boiling in a citric acid repair solution (pH 6.0), the sections were steamed for 3 min, rinsed with tap water, and allowed to cool. Finally, the sections were washed three times in PBS (pH 7.4) for 5 min each. Circles were drawn around the tissue, which was incubated with 3% hydrogen peroxide in the dark for 25 min to block endogenous peroxidase. After washing three times in PBS (pH 7.4), the PBS was blotted off, and 3% BSA serum was applied for blocking using species-specific serum as needed. Blocking was performed for 30 min at room temperature. The blocking solution was blotted off, and the primary antibody working solution was added to each section, which was then incubated overnight at 4 °C in a humidity chamber. The next day, the slides were washed three times in PBS (pH 7.4) for 5 min each. After drying, HRP-conjugated secondary antibody was added and incubated for 1 h at room temperature. Following three washes in PBS, species-specific TSA was added and the slides were incubated in the dark for 10 min. The sections were then heated in an antigen retrieval solution in a microwave at medium power for 8 min, followed by standing for 8 min, and then at medium-low power for 7 min. After antibody incubation, the slides were washed thrice in PBS (pH 7.4) for 5 min each. A fluorescence quenching agent was added and incubated for 5 min, followed by rinsing under running water for 20 min. The sections were mounted with an anti-fade medium and observed under a fluorescence microscope. Photographs were taken and saved if the fluorescence was satisfactory.

### Statistical analysis

IF and cell counting were analyzed using Image J software. Schematic diagrams and illustrations were created using the Adobe Illustrator 2022. Statistical analysis and graphical representations were performed using GraphPad Prism 10.0.2. Data are presented as mean (X̅) ± standard error (SE) from three independent experiments. Regarding the normality tests, all data were first assessed for normality using the Shapiro-Wilk test, implemented in GraphPad Prism 10.0.2. Based on the results, we chose the appropriate statistical tests. Student’s t-test was used for comparisons between two groups, while one-way ANOVA with post-hoc Tukey’s test was applied for comparisons across multiple groups. A P value greater than 0.05 indicated no statistical significance (‘ns’), whereas a *P* value of <0.05 was considered statistically significant, with significance levels represented as ‘*‘, ‘**‘, ‘***‘, and ‘****‘ for *P* values of <0.05, <0.01, <0.001, and <0.0001, respectively.

## Results

### PVL surgery and assessment, exosomes isolation, and identification

In rodents, the liver can regenerate to a size comparable to its original mass within 5–7 days after surgery. Various surgical techniques, resection volumes, and mouse strains lead to different peak proliferation levels, all occurring before 48 h after surgery [29, 30], Thus, 6, 24, and 48 h were selected as observation time points after PVL. Gross observation of the specimen revealed that at 6 h, ischemic changes were noted on the atrophic side, whereas at 24 h, significant ischemic changes and local necrosis were evident. At 48 h, the volume of the atrophic liver decreased, and the area of local necrosis was smaller than that at 24 h, although necrotic foci remained visible (Fig. 1B). H&E Staining revealed extensive necrosis of liver cells around the portal vein at 24 h, with a reduction in necrosis at 48 h, which was replaced by normal cells (Fig. 1C). Ki67 expression was significantly increased in proliferating cells on the hypertrophic side at 24 and 48 h, particularly at 48 h, when numerous Ki67^+^ cells were observed between the central vein and portal areas. In contrast, no significant hepatocyte proliferation was observed in atrophic liver tissue at 24 and 48 h, with only a substantial number of proliferating inflammatory cells present (Fig. 1C).

WB analysis showed that exosomes from different sources may exhibit differences in protein markers; BRL-3A cell supernatant exosomes were positive for CD81, CD63, CD9 and Alix, whereas TSG101 was not expressed. Conversely, liver tissue exosomes were positive for TSG101, CD63, and Alix, but negative for CD9 and CD81. β-Actin was expressed in both types of exosomes, but its expression levels varied (Fig. 1D). TEM revealed that both the cell supernatant and liver tissue exosomes had round or oval membrane structures resembling red blood cells, with distinct white reflective membranes (Fig. 1E). NTA results indicated that the particle size of cell supernatant exosomes was 116.4 ± 57.5 nm, with a concentration of 1.4 × 10^12^ particles/mL, while liver tissue exosomes had a particle size of 139.2 ± 57.2 nm and a concentration of 3.9 × 10^11^ particles/mL (Fig. 1F). DiO-labeled exosomes exhibited green fluorescence and formed granular aggregates on the cell membrane after uptake, with both BRL-3A cells being able to take up exosomes from the supernatant and liver tissue (Fig. 1G). These characterization results indicated that the extracted exosomes met the standards set by the International Society for Extracellular Vesicles [31].

### Liver tissue exosomes-activated YAP-Notch signaling promote liver regeneration

**Fig. 2:**
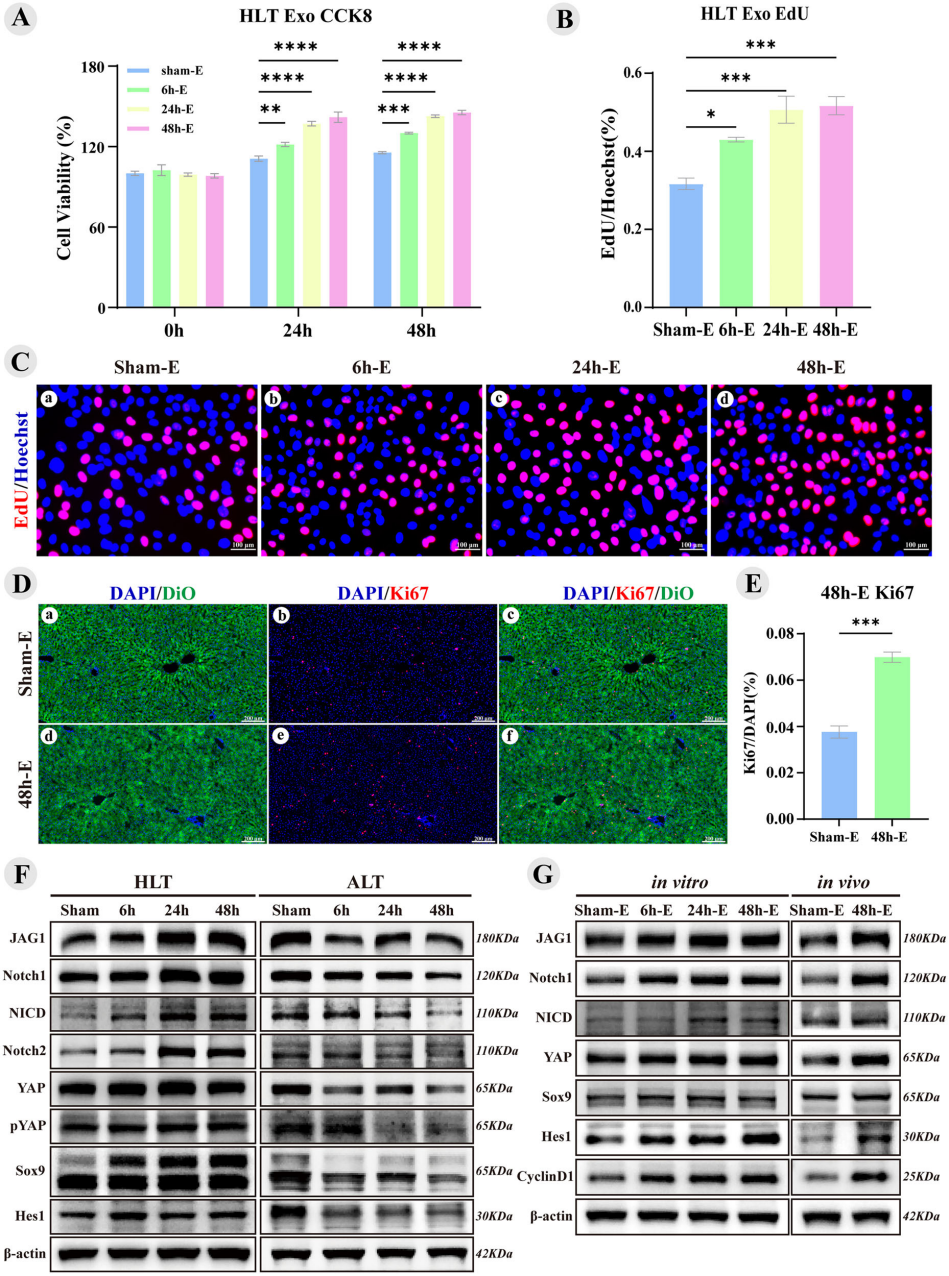
Liver tissue exosomes activated YAP-Notch Signaling promote liver regeneration. Hypertrophic Liver Tissue (HLT), Atrophic Liver Tissue (ALT). Sham-E, 6 h-E, 24 h-E, and 48 h-E refer to exosomes from the hypertrophic side of liver tissue after surgery. **A** Bar chart of CCK8 cell proliferation activity **B** Bar chart depicting the statistical analysis of EdU-positive cells in Fig. C. **C** Images of EdU-positive BRL-3A cells were captured using fluorescence microscopy. **D** Fluorescence images of liver tissue labeled with DiO and Ki67, showing that exosomes are taken up by liver cells, appearing as green fluorescence. **E** Bar chart depicting the statistical analysis of the proliferation index for Ki67-positive cells in Fig. D. **F** Immunoblot bands of YAP-Notch signaling pathway proteins in hypertrophic and atrophic liver tissue after surgery. **G** Immunoblot bands of proteins in the BRL-3A cell line (12 h) and the hypertrophic liver tissue of PVL rats (24 h) after exosome treatment. Values are presented as the mean (X̅) ± standard error (SE), *n* = 3.

In vitro proliferation experiments were performed using the rat BRL-3A liver cell line, and CCK8 and EdU assays were used to evaluate the effects of liver tissue exosomes. Treatment with hypertrophic liver tissue exosomes (6 h-E, 24 h-E and 48 h-E) significantly enhanced cell proliferation compared to Sham-E, with the most pronounced effect observed in the 48 h-E group (Fig. 2A). However, at these three time points, exosomes from the atrophic liver tissue did not promote hepatocyte proliferation (Supplementary Fig. 1). BRL-3A cells treated with hypertrophic liver tissue exosomes (24 h-E and 48 h-E) exhibited increased EdU labeling, and the EdU/Hoechst ratio for the 6 h-E, 24 h-E, and 48 h-E groups were significantly higher than those of Sham-E (Fig. 2B, C). Consequently, 48 h-E was selected for in vivo experiments. In the PVL model, 50 μg of DiO-labeled liver tissue exosomes were injected into the portal vein stump. After 24 h, hypertrophic liver tissues were harvested for IF of Ki67. DiO-labeled exosomes exhibited green fluorescence, confirming liver uptake (Fig. 2D), and the Ki-67/DAPI ratio in the 48 h-E group was significantly higher than that in the Sham-E group.

The YAP-Notch signaling pathway is essential for liver regeneration and plays a significant role in the repair process following liver injury [20, 22, 32, 33]. An increase in nuclear YAP localization are early events following PH in mice [18, 34]. In the early stages of PH, Notch pathway-related genes and proteins, including Notch receptor and ligand JAG1, are significantly upregulated [15, 16]. In hypertrophic liver tissues, RT-qPCR data indicated significant alterations in YAP-Notch-related genes (Supplementary Fig. 2), suggesting their role in liver repair following PVL. WB revealed that the protein expression of JAG1, Notch1, NICD, Notch2, YAP, pYAP, and Sox9 was significantly elevated, whereas Hes1 showed a marked increase at 6 h after surgery, followed by a decline at 24 h. We found that protein expression did not correlate with changes in gene expression, a phenomenon supported by literature on liver regeneration and injury repair [15, 16]. However, this has not been reported in the PVL model, and the specific mechanisms remain unclear. Conversely, protein expression on the atrophic side exhibited an opposite trend compared to that on the hypertrophic side (Fig. 2F). This evidence indicates that the YAP-Notch signaling pathway is activated in the hypertrophic side of the liver.

Liver tissue exosomes may carry substances that activate YAP-Notch signaling pathway-related genes and proteins, contributing to the regeneration of hypertrophic liver after PVL. In vitro treatment of BRL-3A cells with exosomes from hypertrophic liver tissue at 6, 24, and 48 h after PVL resulted in significant upregulation of JAG1, Notch1, NICD, YAP, Sox9, Hes1, and Cyclin D1 compared to Sham-E group (Fig. 2G), and the corresponding gene expression also significantly increased (Supplementary Fig. 3). In vivo treatment of PVL rats with 48h-E also showed significant elevations in these proteins compared to the Sham-E group (Fig. 2G). These findings suggest that exosomes from hypertrophic liver tissue can activate the YAP-Notch signaling pathway both in vitro and in vivo, promoting liver regeneration following PVL.

### Liver tissue exosomes carrying JAG1 activated Notch Signaling promote liver regeneration

**Fig. 3:**
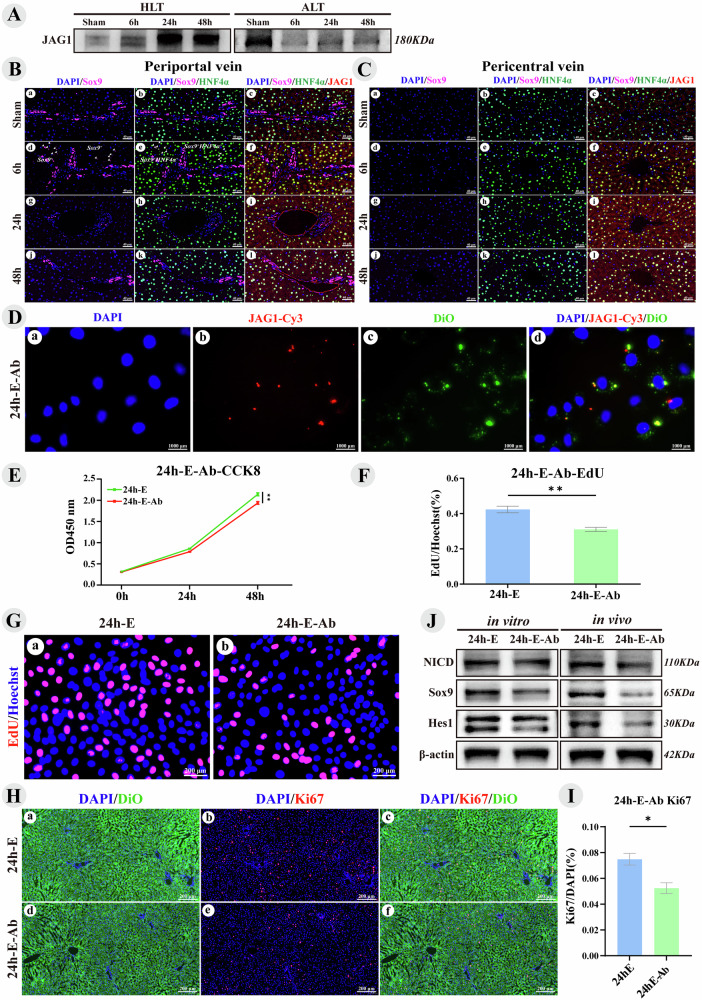
Liver tissue exosomes carrying JAG1 activated Notch Signaling promote liver regeneration. Hypertrophic Liver Tissue (HLT), Atrophic Liver Tissue (ALT), JAG1-Cy3 (exosomes blocked with JAG1 primary antibody and Cy3 fluorescent secondary antibody), 24h-E (PVL24h HLT Exosomes), and 24h-E-Ab (exosomes blocked with JAG1 antibody). **A** Immunoblot bands for JAG1 protein in liver tissue exosomes. **B** and **C** IF of JAG1 protein in the hypertrophic side of the liver tissue, focusing on the periportal and pericentral veins. DAPI (blue) stains nuclei, Sox9 (pink) is a cholangiocyte marker, HNF4α (green) is a hepatocyte marker, and red represents the target protein JAG1. **D** Fluorescent uptake images of JAG1 protein on liver tissue exosomes, captured with a fluorescence microscope, showing exosomes labeled in green (DiO) and red (JAG1-Cy3). **E** Line chart showing CCK8 cell proliferation. **F** Bar chart depicting statistical analysis of EdU-positive cells in Fig. G. **G** Images of EdU-positive BRL-3A cells captured using fluorescence microscopy. **H** Fluorescence images of liver tissues labeled with DiO and Ki67. **I** Bar chart depicting the statistical analysis of the proliferation index of Ki67-positive cells in Fig. H. **J** Immunoblot bands of proteins in the BRL-3A cell line (12 h) and hypertrophic liver tissue of PVL rats (24 h) after exosome treatment. Values are presented as the mean (X̅) ± standard error (SE), *n* = 3.

In vivo and in vitro experiments demonstrated that exosomes from the hypertrophic side of liver tissue activate the YAP-Notch signaling pathway to promote liver regeneration in PVL rats, although the specific cargo and underlying mechanisms remain to be elucidated. The Notch ligand JAG1, which activates Notch through cell-to-cell interactions [35], was elevated in hypertrophic liver tissue after PVL (Fig. 2F). It is still uncertain whether JAG1 also increases in liver exosomes and activates Notch in the adjacent cells. Exosomes from a stiff matrix show elevated JAG1 expression and enhanced tumor proliferation via Notch activation [27]. Furthermore, exosomes from MSCs with stable HIF-1α overexpression increased JAG1 packaging, activated Notch signaling, and induced angiogenesis [26]. WB analysis revealed that JAG1 peaked at 24 h in exosomes from hypertrophic liver tissue, corresponding with tissue changes, whereas its expression in exosomes from the atrophic side declined (Fig. 2F, Fig. 3A). IF analysis demonstrated increased JAG1 in liver tissue, with strong fluorescence in hepatocytes peaking at 24 h (Fig. 3B, C), indicating that liver cells can secrete exosomes carrying JAG1 to facilitate regeneration. Six hours after PVL, Sox9^+^HNF4α^+^ LPCs [22, 36, 37] were identified around the portal vein (Fig. 3B(d–f)), but they were not observed at other time points, which will be further investigated in subsequent sections.

JAG1 on exosomes can be blocked by its antibody [26, 38]. This study used JAG1 antibody (JAG1-Ab) to assess its impact on liver regeneration. The aforementioned research found that exosomes from the hypertrophic side of liver tissue exhibited the highest levels of JAG1 protein 24 h after PVL (Fig. 3A), prompting us to select these exosomes for further investigation. Liver tissue exosomes were labeled green (DiO) and red (JAG1 primary antibody-Cy3 secondary antibody, JAG1-Cy3), confirming that JAG1 antibody could bind to JAG1 on exosomes and be taken up by BRL-3A liver cells (Fig. 3D). In vitro CCK8 assays demonstrated that blocking JAG1 on exosomes reduced liver cell proliferation (Fig. 3E), with EdU assay results showing a significantly lower EdU/Hoechst ratio in the 24 h-E-Ab group compared to the 24 h-E group (Fig. 3F, G). In vivo, a PVL model was established by injecting 50 μg of DiO-labeled 24 h-E and 24 h-E-Ab into the portal vein stump. After 24 h, the harvested hypertrophic liver tissue showed uptake of DiO-labeled exosomes, displaying green fluorescence (Fig. 3H). The Ki-67/DAPI ratio in the 24 h-E-Ab group was significantly lower than in the 24 h-E group (Fig. 3I). In vitro, treatment of BRL-3A cells with 24 h-E-Ab resulted in a significant decrease in the downstream proteins of the Notch signaling pathway (NICD, Sox9, and Hes1), a finding corroborated by in vivo experiments that also showed significant reductions in these proteins compared to the 24 h-E group (Fig. 3J). These findings demonstrate that exosomes from the hypertrophic side of liver tissue can carry JAG1 to activate the Notch signaling pathway, promoting liver regeneration in vitro and in vivo, and this effect was inhibited by the JAG1 antibody.

### ALG-2 promotes the sorting of JAG1 onto exosomes

**Fig. 4:**
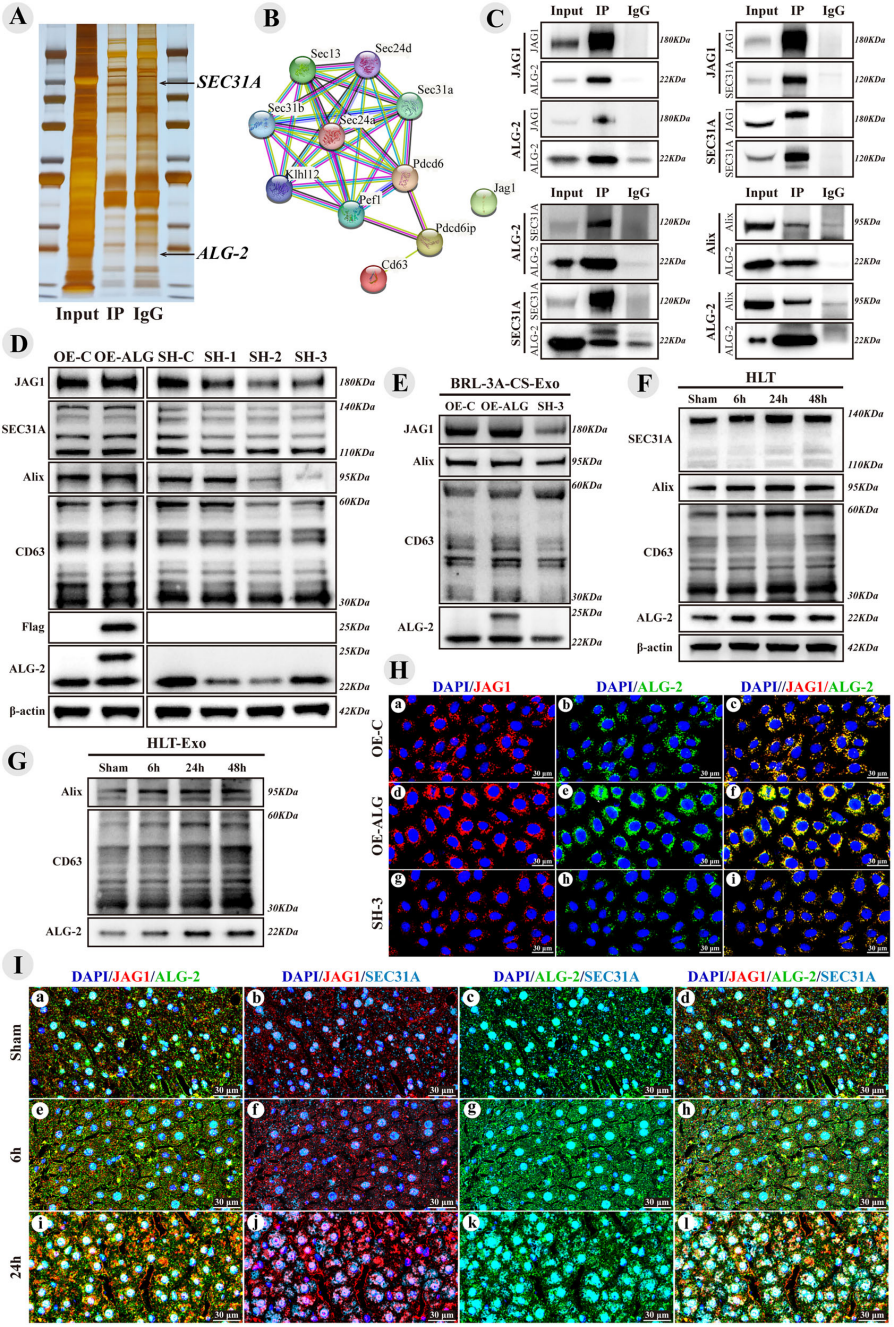
ALG-2 promotes the sorting of JAG1 onto exosomes. Hypertrophic Liver Tissue (HLT), Lentiviral overexpression control (OE-C), Lentiviral overexpression of ALG-2 (OE-ALG), Lentiviral shRNA control (SH-C), Lentiviral shRNA1, shRNA2, and shRNA3 of ALG-2 (SH1, SH2 and SH3). **A** JAG1-IP proteins SDS-PAGE silver staining image. **B** PPI Network from the JAG1-IP mass spectrometry results. **C** Immunoblot bands validating proteins interactions through Co-IP. **D** Immunoblot bands of JAG1 and its interactors in the BRL-3A cell line after lentiviral overexpression and knockdown of ALG-2. **E** Immunoblot bands of JAG1 and its interactors in exosomes from the supernatant of BRL-3A cell line after overexpression and knockdown of ALG-2. **F** Immunoblot bands of JAG1 interactors in hypertrophic liver tissue after surgery. **G** Immunoblot bands of JAG1 interactors in exosomes from hypertrophic liver tissue after surgery. **H** Co-localization immunofluorescent images of JAG1 and ALG-2 in the BRL-3A cell line, with JAG1 in red, ALG-2 in green, and merged fluorescence appearing yellow. **I** Triple co-localization immunofluorescent images of JAG1 (red), ALG-2 (green), and SEC31A (light blue) in hypertrophic liver tissue after surgery.

The mechanisms underlying JAG1 membrane transport and exosome sorting remain unclear, prompting Co-IP experiments targeting JAG1, followed by SDS-PAGE and silver staining, which revealed significant differences in protein bands between the IP and IgG groups (Fig. 4A). Mass spectrometry analysis of immunoprecipitated proteins was performed using STRING for PPI analysis involving SEC31A, PDCD6 (ALG-2), SEC13, Alix (PDCD6IP), CD63, and JAG1 (Fig. 4B), with no current literature documenting the interactions between JAG1 and these proteins. SEC31A, a component of coat protein complex II (COPII), facilitates transport vesicle formation in the endoplasmic reticulum (ER) by deforming the ER membrane and selectively packaging cargo molecules [39, 40]. ALG-2 interacts with SEC31A [41], which is essential for COPII-mediated ER-to-Golgi transport and plays a critical role in endosomal biogenesis and membrane repair [42, 43]. Additionally, ALG-2 enhances ER-Golgi transport by facilitating the interaction between Alix and TSG101, thereby linking the ESCRT-III and ESCRT-I complexes [42, 44]and activating the multivesicular endosome (MVE) sorting function [45]. Alix-dependent ESCRT-III regulates cargo sorting into exosomes by recruiting it to late endosomal membranes, which is crucial for exosome secretion [46, 47]. SDS-PAGE analysis of JAG1 immunoprecipitated proteins revealed potential direct interactions between JAG1 and SEC31A, as well as between JAG1 and ALG-2, with no direct interaction with Alix detected. Co-IP experiments confirmed the interactions between JAG1/ALG-2, JAG1/SEC31A, and ALG-2/SEC31A, and further validated the ALG-2/Alix interaction via Alix Co-IP (Fig. 4C), which also showed no direct interaction with JAG1. These findings suggest that ALG-2 may serve as a crucial molecular bridge between SEC31A and Alix, influencing exosome release and cargo sorting.

Thus, ALG-2 was identified as a key molecule that influences JAG1 sorting onto exosomes. Lentiviruses containing a full-length Flag-tagged ALG-2 sequence were used to infect BRL-3A cell lines, significantly increasing JAG1, SEC31A, Alix, and CD63 protein levels compared to controls, whereas ALG-2 silencing with shRNA decreased these proteins (Fig. 4D). Overexpression of ALG-2 elevated JAG1 levels on exosomes, along with Flag-tagged ALG-2, and increased Alix and CD63, whereas ALG-2 silencing reduced their expression (Fig. 4E). Immunofluorescence co-localization analysis confirmed the co-localization of JAG1 and ALG-2, showing enhanced signals upon ALG-2 overexpression and diminished signals after knockdown, with merged fluorescence channels displaying a yellow signal (Fig. 4H).

After PVL, SEC31A, Alix, CD63, and ALG-2 proteins increased in the hypertrophic side of the liver tissue, peaking at 24 h, which aligned with JAG1 expression (Fig. 4F). In exosomes from this hypertrophic tissue, Alix, CD63, and ALG-2 showed similar trends, with peaks at 24 h (Fig. 4G). Immunofluorescence co-localization revealed that JAG1, ALG-2, and SEC31A colocalized at various time points, most notably at 24 h after surgery. JAG1 and ALG-2 colocalized in both the cytoplasm and cell membrane, whereas JAG1/SEC31A and ALG-2/SEC31A co-localization was mainly cytoplasmic, particularly pronounced at 24 h after PVL (Fig. 4I). These findings suggest that ALG-2 and SEC31A are key proteins that facilitate the intracellular transport and sorting of JAG1 onto exosomes.

### YAP induces exosomes carrying JAG1-activated Notch Signaling promote liver regeneration

**Fig. 5:**
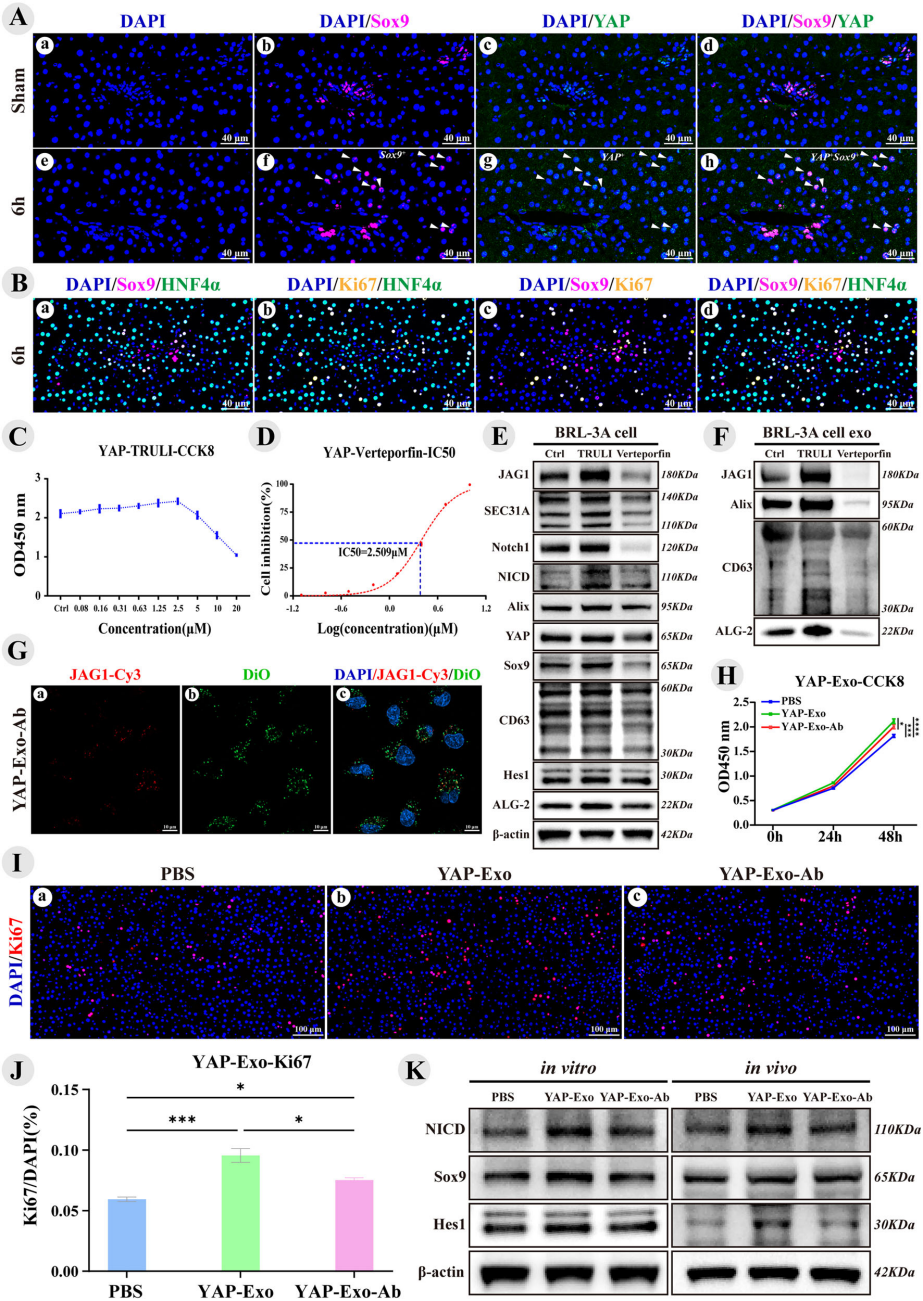
YAP induces exosomes carrying JAG1 activated Notch Signaling promote liver regeneration. YAP-Exo refers to exosomes from the supernatant of BRL-3A cells treated with 2.5 μM TRULI, while YAP-Exo-Ab indicates YAP-Exo after blockade with JAG1 antibody. JAG1-Cy3 (Exosomes blocked with JAG1 primary antibody and Cy3 fluorescent secondary antibody). DAPI (blue) stains cell nuclei, Sox9 (pink) is a cholangiocyte-specific marker, HNF4α (green) is a hepatocyte-specific marker, and Ki67 (yellow) marks proliferating cells. **A** Sox9/YAP dual-IF images reveal YAP-positive nuclei in Sox9^+^ LPCs around the portal vein. **B** Sox9/Ki67/HNF4α triple-IF images show that (**a**) Sox9^+^ cells are hepatocytes, (**b**) proliferating cells are also hepatocytes, (**c**) Sox9^+^ cells are non-proliferating, and (**d**) the merged fluorescence image reveals that Sox9^+^HNF4α^+^ LPCs are non-proliferating hepatocytes. **C** Cell proliferation curves after treatment with various doses of TRULI. **D** IC50 curve of Verteporfin **E** Immunoblot bands in the BRL-3A cell line after treatment with 2.5 μM TRULI and Verteporfin. **F** Immunoblot bands in the supernatant exosomes of the BRL-3A cell line after treatment with 2.5 μM TRULI and Verteporfin. **G** Fluorescent uptake images of JAG1 protein on YAP-Exo, captured with a confocal fluorescence microscope, show exosomes labeled in green (DiO) and red (JAG1-Cy3). **H** Line chart of CCK8 cell proliferation activity after treatment with PBS, YAP-Exo, and YAP-Exo-Ab. **I** Fluorescence images of liver tissue labeled with Ki67 after treatment with PBS, YAP-Exo, and YAP-Exo-Ab. **J** Bar chart depicting the statistical analysis of the proliferation index for Ki67-positive cells in Fig. J. **K** Immunoblot bands in the BRL-3A cell line (12 h) and the hypertrophic liver tissue of PVL rats (24 h) after treatment with PBS, YAP-Exo, and YAP-Exo-Ab. Values are presented as the mean (X̅) ± standard error (SE), *n* = 3.

Sox9 is a marker for LPCs and cholangiocytes, acting as a downstream target of YAP activation that transforms mature hepatocytes into Sox9^+^HNF4α^+^ LPCs [22, 24, 32, 36, 48]. Following PVL, Sox9^+^HNF4α^+^ cells were observed around the portal vein at 6 h (Fig. 3Bd–f), although their functions remain unclear. Dual IF revealed nuclear YAP positivity in Sox9^+^ hepatocytes, while the Sham group showed no significant fluorescence (Fig. 5A). Triple IF with Ki67, Sox9, and HNF4α indicated that these Sox9^+^HNF4α^+^ cells were non-proliferative (Fig. 5B), likely resulting from YAP-induced reprogramming. Additionally, extensive crosstalk exists between the Hippo-YAP and Notch pathways, with JAG1 acting as a direct target of YAP-TEAD [21, 34, 49, 50]. Further research is necessary to ascertain whether exosomes from YAP-induced Sox9^+^HNF4α^+^ LPCs can transport JAG1 to activate the Notch signaling pathway in neighboring cells, thereby facilitating liver regeneration.

TRULI is a Lats kinase inhibitor that suppresses YAP phosphorylation and, positively regulates YAP/TAZ activity [51]. Verteporfin, a YAP inhibitor, disrupts the YAP-TEAD interaction and inhibits downstream gene expression [52]. In BRL-3A cells, the optimal concentration for TRULI was 2.5 μM (Fig. 5C), while Verteporfin had an IC50 of 2.509 μM (Fig. 5D). TRULI promoted hepatocyte proliferation；however, excessive doses increased toxicity and inhibited growth, whereas Verteporfin significantly suppressed proliferation (Fig. 5C, D). Treatment with 2.5 μM TRULI increased the expression of Notch signaling proteins (JAG1, Notch1, NICD, Sox9, and Hes1), JAG1 interactors (SEC31A/ALG-2), and exosome secretion proteins (Alix/CD63), while 2.5 μM Verteporfin decreased these levels (Fig. 5E), suggesting YAP acted upstream of Notch. YAP activity also influenced Sox9 expression (Fig. 5E) and combined with the YAP and Sox9 dual-IF results (Fig. 5A), suggesting that Sox9^+^HNF4α^+^ LPCs may arise from YAP-induced reprogramming. YAP activation promoted JAG1, Alix, CD63, and ALG-2 expression in exosomes, whereas YAP inhibition decreased their levels (Fig. 5F). Coupled with changes in JAG1 interactors (SEC31A/ALG-2) in cell lines (Fig. 5E), this suggests that YAP regulats JAG1 expression on exosomes via the SEC31A/ALG-2/Alix axis, indicating that YAP^+^Sox9^+^HNF4α^+^ LPCs may play a crucial role in liver regeneration after PVL and serve as a source of JAG1^+^ exosomes.

Exosomes from hepatocytes treated with 2.5 μM TRULI (YAP-Exo) showed that DiO (green) and JAG1-Ab-Cy3 (red) staining confirmed JAG1 antibody binding to JAG1 on YAP-Exo, which was subsequently taken up by BRL-3A hepatocytes (Fig. 5G). CCK8 assays indicated that YAP-Exo promoted BRL-3A cell proliferation, whereas blocking with the JAG1 antibody weakened this effect (Fig. 5H). In vivo, in the PVL model, the portal vein stump was injected with 50 μg YAP-Exo and YAP-Exo-Ab, with the control group receiving PBS. After 24 h, the harvested liver tissue showed a significant increase in the Ki-67/DAPI ratio in the YAP-Exo group, which decreased upon blocking with JAG1 antibody (Fig. 5I, J). In vitro, YAP-Exo treatment elevated NICD, Sox9, and Hes1 expression compared to PBS, whereas JAG1 blockade reduced these proteins, consistent with the in vivo results (Fig. 5K). Overall, YAP may induce hepatocyte reprogramming to Sox9^+^HNF4α^+^ LPCs and release JAG1^+^ exosomes via the SEC31A/ALG-2/Alix axis, activating the Notch signaling pathway in neighboring cells to promote liver regeneration in PVL rats.

### Mechanism diagram of this study

**Fig. 6:**
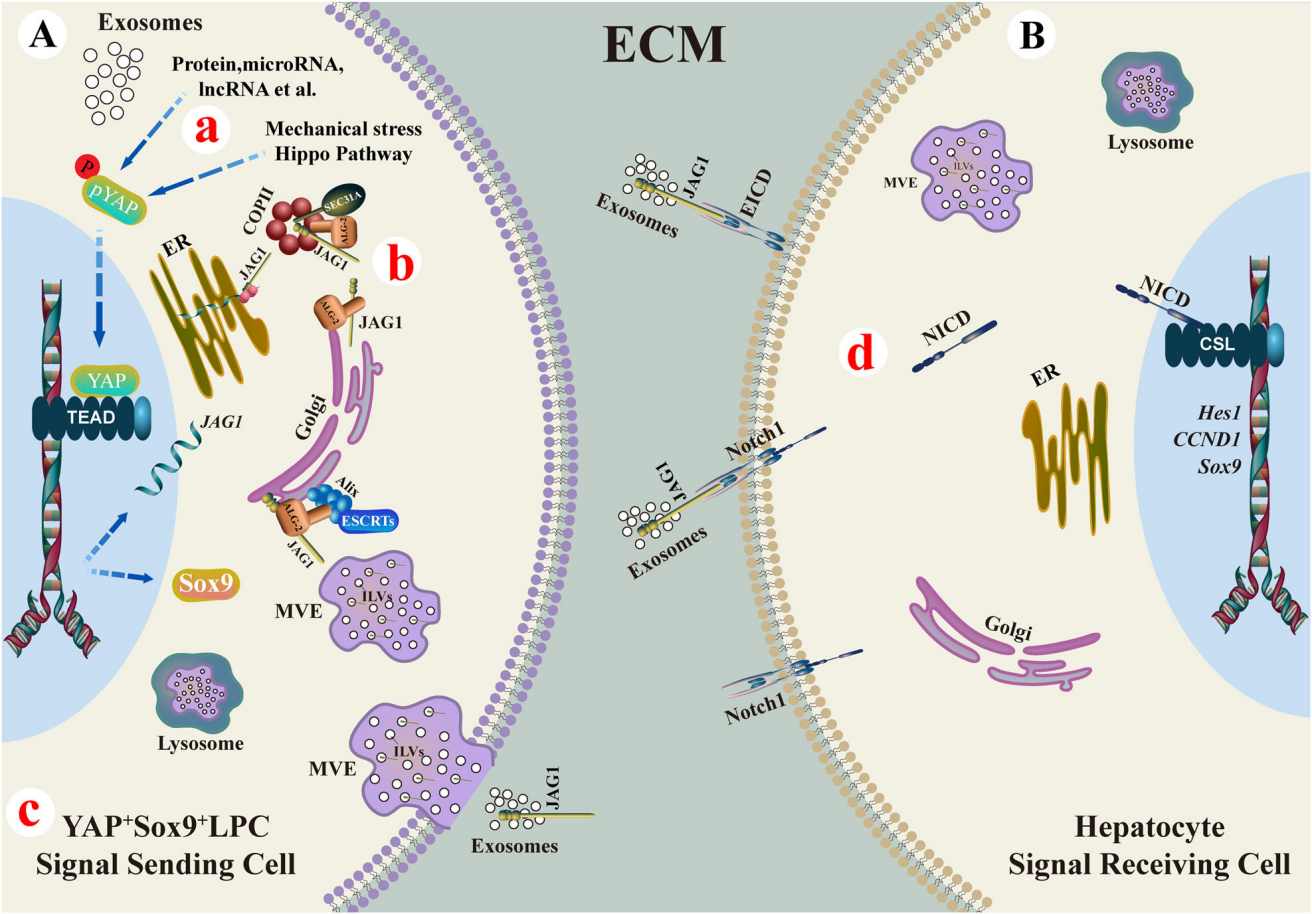
Mechanism Diagram of This Study. **A** Signal Sending Cell (YAP^+^Sox9^+^LPC). **B** Signal Receiving Cell (Hepatocyte). Extracellular matrix (ECM), Liver progenitor cells (LPCs), Endoplasmic reticulum (ER), Golgi apparatus (Golgi), Multivesicular endosome (MVE), Intraluminal vesicles (ILVs), Coat protein II (COPII). Created with the Adobe Illustrator 2022.

1. YAP-Notch activation was confirmed in hypertrophic liver tissues, along with the finding that liver-derived exosomes activated this pathway, promoting liver regeneration (Fig. 6a). 2. Co-IP-MS combined with Co-IP identified interactions between JAG1/ALG-2, JAG1/SEC31A, ALG-2/SEC31A, and ALG-2/Alix, indicating that ALG-2 serves as a key connector between SEC31A and Alix, facilitating JAG1 sorting on exosomes (Fig. 6b). 3. YAP reprogrammed hepatocytes to Sox9^+^HNF4α^+^ LPCs (Fig. 6c) and promoted the release of JAG1^+^ exosomes via the SEC31A/ALG-2/Alix axis (Fig. 6b), suggesting that YAP^+^Sox9^+^ LPCs are a source of JAG1^+^ exosomes. 4. YAP-induced liver cell exosomes carrying JAG1 activated the Notch signaling pathway in neighboring hepatocytes, enhancing liver regeneration (Fig. 6d).

## Discussion

Surgeons primarily focus on preoperative strategies to promote liver regeneration and address postoperative liver failure, along with the mechanisms of regeneration after surgery. Most studies have focused on the PH model, while research on the PVL model is limited. PVL/PVE has been clinically shown to increase liver volume [4, 29], making it an acceptable approach for achieving sufficient residual liver volume and reducing perioperative risks, thereby enhancing the resectability of large liver cancers [53, 54]. Using H&E staining and Ki67 immunohistochemistry, we characterized liver repair after PVL. Significant hepatocyte proliferation was observed on the hypertrophic side, peaking at 48 h and primarily located in zone 2 [1]. These findings are consistent with the reported proliferation patterns following PH [55, 56]. In contrast, the atrophic side showed a substantial volume reduction, with extensive necrosis noted at 24 h, particularly around the portal vein. Notably, abundant inflammatory cell infiltration was observed in this region at 24 h, while Ki67 revealed no significant hepatocyte proliferation.

Increasing research on exosomes has highlighted their crucial role as regulators of homeostasis in healthy individuals and in disease pathology [57–59]. Exosomes from both normal and damaged liver tissues promote proliferation following liver injury [13]. Hepatocyte-derived exosomes can lead to a dose-dependent increase in hepatocyte proliferation, whereas exosomes from Kupffer cells and LSECs do not induce this effect [12]. Treatment of human primary hepatocytes with components such as A83-01, CHIR99021, and HGF can reprogram them into bipotential human LPCs, whose derived exosomes support the survival of damaged hepatocytes and promote liver regeneration [60]. DiO fluorescence demonstrated that liver tissue exosomes could be taken up by hepatocytes both in vivo and in vitro. Exosomes from the hypertrophic side at 6, 24, and 48 h after PVL enhanced hepatocyte proliferation, with the most significant effect observed at 48h-E, whereas exosomes from the atrophic side did not promote proliferation.

The YAP-Notch signaling pathway is essential for liver regeneration and significantly contributes to the repair process following liver injury [15, 16, 22, 32, 33]. Extracellular matrix stiffness is closely linked to the Hippo-YAP signaling pathway[61–63]. After surgery, immediate alterations in intrahepatic blood flow increase mechanical stress on the liver, activate mechanoreceptors that modulate Hippo-YAP signaling and regulate hepatocyte proliferation during liver regeneration [62, 63]. The Hippo-YAP and Notch pathways exhibit considerable crosstalk, with Notch receptors and ligands serving as direct downstream targets of YAP-TEAD [21, 34, 64]. Inducing YAP bioactivity in vivo activates Notch and reprograms hepatocytes into liver progenitor cells [23]. In normal mouse hepatocyte lines, shRNA knockdown of YAP downregulates Notch1 signaling, whereas YAP overexpression enhances Notch1-NICD signaling [20]. Furthermore, in HCC cells and mouse hepatocytes, upregulation of JAG1 activates Notch signaling, promoting cell and tumor proliferation [21]. Our study found that genes and proteins in the YAP-Notch pathway were activated after PVL, exhibiting asynchronous change trends—a phenomenon supported by the existing literature in PH [15, 16]—yet no reports in the PVL model, leaving the reasons unclear and warranting further investigation. Additionally, we demonstrated that exosomes from the hypertrophic side could activate the YAP-Notch pathway, promoting liver regeneration after PVL.

Sheldon et al. [65] first reported that the Notch ligand Dll4 can be packaged into exosomes. Exosomes from a stiff matrix exhibited elevated JAG1 expression and enhanced tumor proliferation through Notch activation [27]. Moreover, exosomes derived from MSCs with stable HIF-1α overexpression increased JAG1 packaging, activated Notch signaling, and promoted angiogenesis [26]. Although JAG1 activates Notch through cell-to-cell interactions [35], it remains uncertain whether JAG1 levels increase on liver exosomes to activate Notch in adjacent cells. JAG1 on exosomes can be blocked by an antibody [26, 38]. Through DiO and JAG1-Cy3 fluorescence uptake experiments, we confirmed that the JAG1 antibody bound to JAG1 on exosomes and was taken up by liver cells. Our findings demonstrate that exosomes from the hypertrophic side of liver tissue carry JAG1, activate the Notch signaling pathway, and promote liver regeneration in vitro and in vivo, which can be inhibited by the JAG1 antibody.

However, the mechanisms underlying JAG1 membrane transport and exosome sorting remain unclear. Exosome formation involves double invagination of the plasma membrane, resulting in the generation of intraluminal vesicles (ILVs) within MVEs, which release exosomes into the extracellular space upon fusion with the plasma membrane [66]. While ESCRT-III is essential for the entry of ILVs into the lumen of MVEs, cargo aggregation and membrane budding can occur via both ESCRT-dependent and independent mechanisms [47]. Membrane cargo is sorted from the Golgi to the endosomes or internalized from the plasma membrane, with ILVs packaging occurring during endosome maturation. The key players in this process include Rab GTPases, Alix, and CD63 [66, 67]. Co-IP-MS combined with Co-IP identified interactions between JAG1/ALG-2, JAG1/SEC31A, ALG-2/SEC31A, and ALG-2/Alix. SEC31A, a component of COPII, facilitates vesicle formation in the endoplasmic reticulum (ER) by deforming the ER membrane and selectively packaging cargo molecules [39, 40]. ALG-2 interacts with SEC31A [41], which is essential for COPII-mediated ER-to-Golgi transport and plays a critical role in endosomal biogenesis and membrane repair [42, 43]. Additionally, ALG-2 enhances ER-Golgi transport by facilitating the interaction between Alix and TSG101, thereby linking the ESCRT-III and ESCRT-I complexes [42, 44] and activating the MVE sorting function [45]. Alix-dependent ESCRT-III regulates cargo sorting into exosomes by recruiting it to late endosomal membranes, which is crucial for exosome secretion [46, 47]. Thus, ALG-2 was identified as a key molecule that influences JAG1 sorting onto exosomes. We validated the expression of JAG1, SEC31A, ALG-2, Alix, and CD63 in cell lines and their exosomes through ALG-2 overexpression and knockdown, and confirmed their expression in liver tissues and liver-derived exosomes. Combined with triple IF of JAG1, SEC31A, and ALG-2, we demonstrated that ALG-2 serves as a key protein linking SEC31A and Alix, facilitating the sorting of JAG1 onto exosomes and elucidating the mechanism of JAG1 exosome sorting.

Sox9, a marker for LPCs and cholangiocytes, acts as a downstream target of YAP activation, enabling mature hepatocytes to transform into Sox9^+^HNF4α^+^ LPCs, which can subsequently redifferentiate into hepatocyte and cholangiocyte lineages [22, 24, 32, 34, 36, 68]. Following PVL, Sox9^+^HNF4α^+^ cells were observed around the portal vein at 6 h. Dual-IF revealed nuclear YAP positivity in Sox9^+^ hepatocytes, whereas triple IF of Ki67, Sox9, and HNF4α indicated that these Sox9^+^HNF4α^+^ cells were non-proliferative, likely due to YAP-induced reprogramming of LPCs. Additionally, extensive crosstalk exists between the Hippo-YAP and Notch pathways, with JAG1 serving as a direct target of YAP-TEAD [21, 34, 49, 50]. Our experiments demonstrated that YAP^+^Sox9^+^ LPCs may be a source of JAG1^+^ exosomes and that YAP-induced hepatocytes can release JAG1^+^ exosomes via the SEC31A/ALG-2/Alix axis, thereby activating the Notch signaling pathway in neighboring cells to promote liver regeneration in PVL rats. While the precise lineage fate of Sox9⁺HNF4α⁺ cells remains to be fully defined, our data suggest that they represent a reprogrammed intermediate state rather than fully committed LPCs. Their primary role may not be direct proliferation, but rather functional signaling—such as releasing JAG1⁺ exosomes to activate Notch in surrounding hepatocytes, as demonstrated in our study. Therefore, we propose that Sox9⁺HNF4α⁺ cells act as a transient but functionally important cell population, serving as both responders to mechanical/YAP activation and initiators of local regeneration via exosome-mediated communication.

Overall, we demonstrated that after PVL surgery, liver tissue produced significant amounts of exosomes released into the interstitial space, activating the YAP-Notch signaling pathway to promote liver regeneration. YAP^+^Sox9^+^ LPCs may have served as a source of JAG1^+^ exosomes, which activated Notch signaling in neighboring hepatocytes and enhanced regeneration in PVL rats. Our findings suggest that JAG1⁺ exosomes derived from regenerating liver tissue may serve as a promising cell-free therapeutic strategy to promote liver regeneration, particularly in clinical contexts such as acute liver injury, post-hepatectomy liver failure, and liver transplantation. Compared to traditional stem cell therapies, exosomes offer several translational advantages, including lower immunogenicity, easier storage, and greater potential for large-scale production. However, future research must address key challenges before clinical application can be realized. These include improving delivery specificity to injured hepatic tissue, standardizing isolation and purification protocols to ensure batch consistency, and conducting long-term safety and biodistribution studies to evaluate potential immunogenicity and off-target effects. Addressing these issues will be critical for developing exosome-based therapies that are safe, effective, and clinically scalable.

This study has limitations because the complexity of the YAP-Notch signaling pathway involves many key upstream and downstream proteins. We focused on the core membrane protein JAG1 without performing omics analysis on exosomes to identify additional critical molecules. In future research, we will conduct an omics analysis of exosomes to further explore the mechanisms of the YAP-Notch signaling pathway in liver regeneration, aiming to provide more comprehensive theoretical support for both basic research and clinical applications.

## Supplementary information

Antibodies

Primer sequences

Supplementary Figures

Uncropped western blots

## Data Availability

The datasets generated and analyzed during this study are available from the corresponding author upon reasonable request.
