# Supplementary material for: Exosomes from liver progenitor cells carrying JAG1 activate notch signaling to promote liver regeneration in PVL rats
Source: Cell Death Dis. 2025 Aug 12;16(1):609. doi: 10.1038/s41419-025-07925-1 (PMC12343779; doi:10.1038/s41419-025-07925-1)
Supplement: Supplementary file 1 — Antibodies [file 41419_2025_7925_MOESM1_ESM.docx]

| **Antibodies** | **Brands** | **Catalog numbers** |
| --- | --- | --- |
| JAG1 primary antibody (rabbit) | Abcam | ab300561 |
| JAG1 primary antibody (mouse) | [Santa Cruz Biotechnology](https://www.baidu.com/link?url=XWtXDzm-f5wAbp-b5jlC51XAxuGIOQu-SsdLzbGAVBi4omJHRIABw_PZigo-5Ax_&wd=&eqid=f9502a1c019330d20000000365c859f1) | sc-390177 |
| Notch1 primary antibody (rabbit) | CST | 3608T |
| NICD primary antibody (rabbit) | CST | 4147T |
| Notch2 primary antibody (mouse) | [Santa Cruz Biotechnology](https://www.baidu.com/link?url=XWtXDzm-f5wAbp-b5jlC51XAxuGIOQu-SsdLzbGAVBi4omJHRIABw_PZigo-5Ax_&wd=&eqid=f9502a1c019330d20000000365c859f1) | [sc-518169](https://www.scbt.com/zh/p/notch-2-antibody-f-10?requestFrom=search) |
| YAP primary antibody (mouse) | [Santa Cruz Biotechnology](https://www.baidu.com/link?url=XWtXDzm-f5wAbp-b5jlC51XAxuGIOQu-SsdLzbGAVBi4omJHRIABw_PZigo-5Ax_&wd=&eqid=f9502a1c019330d20000000365c859f1) | [sc-101199](https://www.scbt.com/zh/p/yap-antibody-63-7?requestFrom=search) |
| YAP primary antibody (rabbit) | CST | 14074T |
| pYAP primary antibody (rabbit) | Affinity Biosciences | [AF3328](https://m.affbiotech.cn/goods-1078-AF3328-Phospho_YAP_Ser127_Antibody.html) |
| Sox9 primary antibody (rabbit) | Sigma-Aldrich | AB5535 |
| Hes1 primary antibody (rabbit) | Abcam | ab108937 |
| CyclinD1 primary antibody (mouse) | Proteintech | 60186-1-Ig |
| Alix primary antibody (mouse) | [Santa Cruz Biotechnology](https://www.baidu.com/link?url=XWtXDzm-f5wAbp-b5jlC51XAxuGIOQu-SsdLzbGAVBi4omJHRIABw_PZigo-5Ax_&wd=&eqid=f9502a1c019330d20000000365c859f1) | sc-53540 |
| CD63 primary antibody (mouse) | [Santa Cruz Biotechnology](https://www.baidu.com/link?url=XWtXDzm-f5wAbp-b5jlC51XAxuGIOQu-SsdLzbGAVBi4omJHRIABw_PZigo-5Ax_&wd=&eqid=f9502a1c019330d20000000365c859f1) | sc-5275 |
| Tsg101 primary antibody (mouse) | [Santa Cruz Biotechnology](https://www.baidu.com/link?url=XWtXDzm-f5wAbp-b5jlC51XAxuGIOQu-SsdLzbGAVBi4omJHRIABw_PZigo-5Ax_&wd=&eqid=f9502a1c019330d20000000365c859f1) | sc-7964 |
| CD9 primary antibody (mouse) | [Santa Cruz Biotechnology](https://www.baidu.com/link?url=XWtXDzm-f5wAbp-b5jlC51XAxuGIOQu-SsdLzbGAVBi4omJHRIABw_PZigo-5Ax_&wd=&eqid=f9502a1c019330d20000000365c859f1) | sc-13118 |
| CD81 primary antibody (mouse) | [Santa Cruz Biotechnology](https://www.baidu.com/link?url=XWtXDzm-f5wAbp-b5jlC51XAxuGIOQu-SsdLzbGAVBi4omJHRIABw_PZigo-5Ax_&wd=&eqid=f9502a1c019330d20000000365c859f1) | sc-166029 |
| Ki67 primary antibody (rabbit) | Abcam | ab16667 |
| Ki67 primary antibody (rabbit) | Wuhan Servicebio Technology Co., Ltd. | GB111499-100 |
| Sec31A primary antibody (rabbit) | Proteintech | 17913-1-AP |
| ALG-2 primary antibody (rabbit) | Proteintech | 12303-1-AP |
| ALG-2 primary antibody (mouse) | [Santa Cruz Biotechnology](https://www.baidu.com/link?url=XWtXDzm-f5wAbp-b5jlC51XAxuGIOQu-SsdLzbGAVBi4omJHRIABw_PZigo-5Ax_&wd=&eqid=f9502a1c019330d20000000365c859f1) | sc-376950 |
| Flag primary antibody (mouse) | Shanghai ShareBio Technology Co., Ltd. | SB-AB0008 |
| β-actin（Directly conjugated primary antibodies） | Shanghai ShareBio Technology Co., Ltd. | SB-AB2001 |
| Alexa Fluor 488-conjugated goat anti-rabbit IgG | Wuhan Servicebio Technology Co., Ltd. | GB25303 |
| Alexa Fluor 488-conjugated goat anti-mouse IgG | Wuhan Servicebio Technology Co., Ltd. | GB25301 |
| CY5 goat anti-mouse IgG | Wuhan Servicebio Technology Co., Ltd. | GB27301 |
| CY5 goat anti-rabbit IgG | Wuhan Servicebio Technology Co., Ltd. | GB27303 |
| Cy3 goat anti-rabbit IgG | Wuhan Servicebio Technology Co., Ltd. | GB21303 |
| Cy3 goat anti-mouse IgG | Wuhan Servicebio Technology Co., Ltd. | GB21301 |
| [HRP goat anti-rabbit IgG](https://store.sangon.com/productDetail?productInfo.code=D110058) | Sangon Biotech (Shanghai) Co., Ltd. | [D110058](https://store.sangon.com/productDetail?productInfo.code=D110058) |
| [HRP goat anti-mouse IgG](https://store.sangon.com/productDetail?productInfo.code=D110087) | Sangon Biotech (Shanghai) Co., Ltd. | [D110087](https://store.sangon.com/productDetail?productInfo.code=D110087) |
