# Supplementary material for: Exosomes from liver progenitor cells carrying JAG1 activate notch signaling to promote liver regeneration in PVL rats
Source: Cell Death Dis. 2025 Aug 12;16(1):609. doi: 10.1038/s41419-025-07925-1 (PMC12343779; doi:10.1038/s41419-025-07925-1)
Supplement: Supplementary file 2 — Primer sequences [file 41419_2025_7925_MOESM2_ESM.docx]

| **Genes** | **Sequences** |
| --- | --- |
| *JAG1* sense | AACTGGTACCGGTGCGAA |
| *JAG1* anti-sense | TGATGCAAGATCTCCCTGAAAC |
| *Notch1* sense | ATGATGGCACAACCCCTCTG |
| *Notch1* anti-sense | CCAGCAACACTTTGGCAGTC |
| *YAP* sense | AGACGACTTCCTGAACAGCG |
| *YAP* anti-sense | GTGCCAAGGTCCACATTTG |
| *Wwtr1（TAZ）*sense | ATCATTCACGGGAGCAGAG |
| *Wwtr1（TAZ）*anti-sense | CACCTGTGTCCATCTCATCC |
| *Notch2* sense | AGTGGTATGGACTGTGAGGAGG |
| *Notch2* anti-sense | CAGGAGAAGGTGTTCACTTTGTC |
| *Hes1* sense | CAACACGACACCGGACAAAC |
| *Hes1* anti-sense | GGAATGCCGGGAGCTATCTT |
| *Cyr61* sense | TGTTGAGCATCGTGGAGAC |
| *Cyr61* anti-sense | ACTGCGACTGCGTTACTGTC |
| *Hey1* sense | AATGCCTGGCTGAAGTTG |
| *Hey1* anti-sense | GGATGCGTAGTTGTTGAGATG |
| *Sox9* sense | TGACTACACCGACCACCAGA |
| *Sox9* anti-sense | GAGCTGTGTGTAGACGGGTT |
| *CCND1* sense | TGGAACTGCTTCTGGTGAAC |
| *CCND1* anti-sense | AAAGTGCGTTGTGCGGTAG |
