# Supplementary material for: Exosomes from liver progenitor cells carrying JAG1 activate notch signaling to promote liver regeneration in PVL rats
Source: Cell Death Dis. 2025 Aug 12;16(1):609. doi: 10.1038/s41419-025-07925-1 (PMC12343779; doi:10.1038/s41419-025-07925-1)
Supplement: Supplementary file 3 — Supplementary Figures [file 41419_2025_7925_MOESM3_ESM.docx]

**
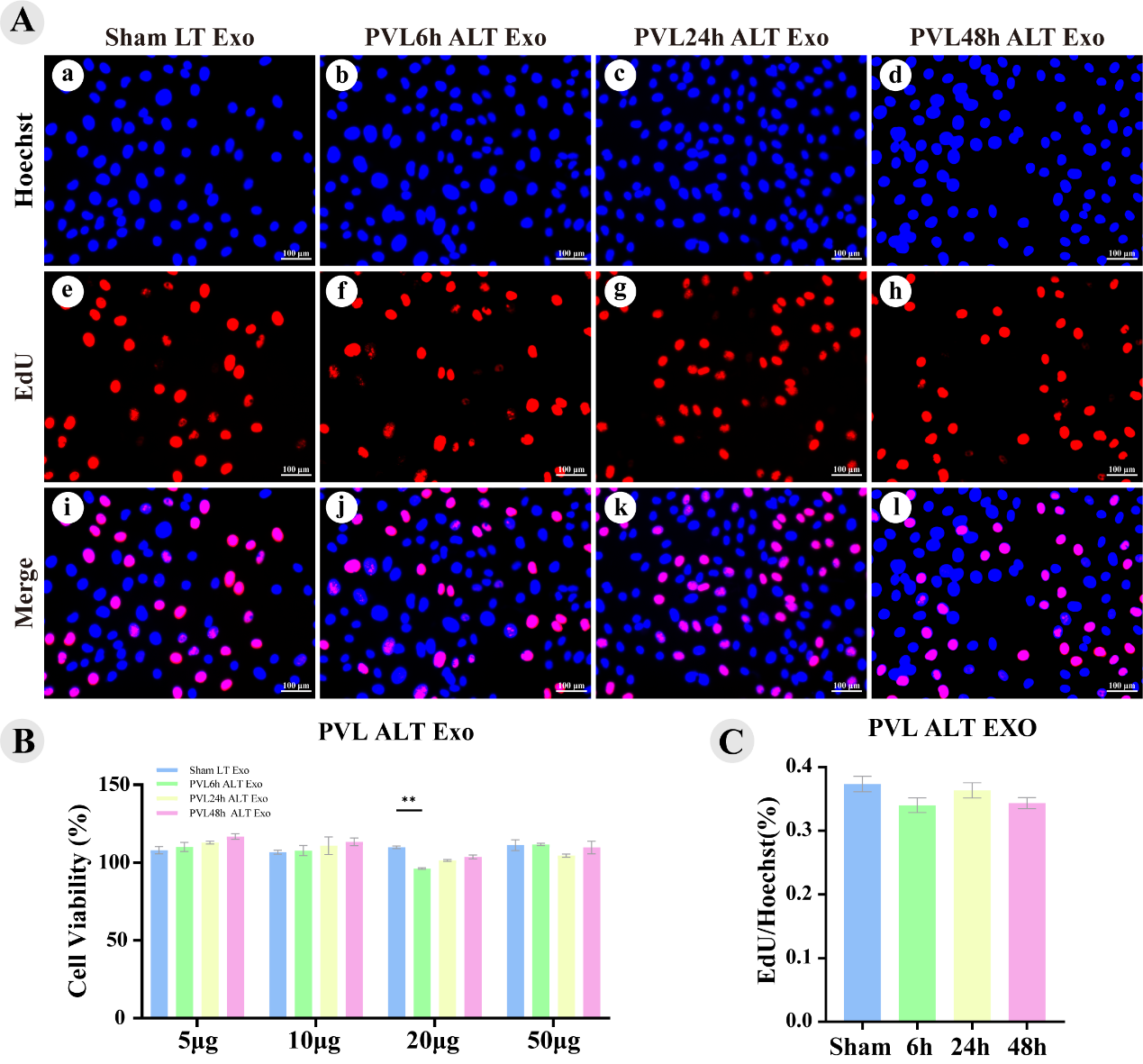
Supplementary Fig.1 Exosomes derived from atrophic liver tissue were unable to promote liver regeneration.**

Atrophic Liver Tissue (ALT). Sham LT Exo, PVL6h ALT Exo, PVL24h ALT Exo, and PVL48h ALT Exo refer to exosomes from the atrophic side of liver tissue after surgery. **(A)**, Images of EdU-positive BRL-3A cells were captured using fluorescence microscopy. **(B)**, Bar chart of CCK8 cell proliferation activity. **(C)**, Bar chart depicting the statistical analysis of EdU-positive cells in Fig. A. Values are presented as the mean (X̅) ± standard error (SE), n=3.

**Supplementary Fig.**
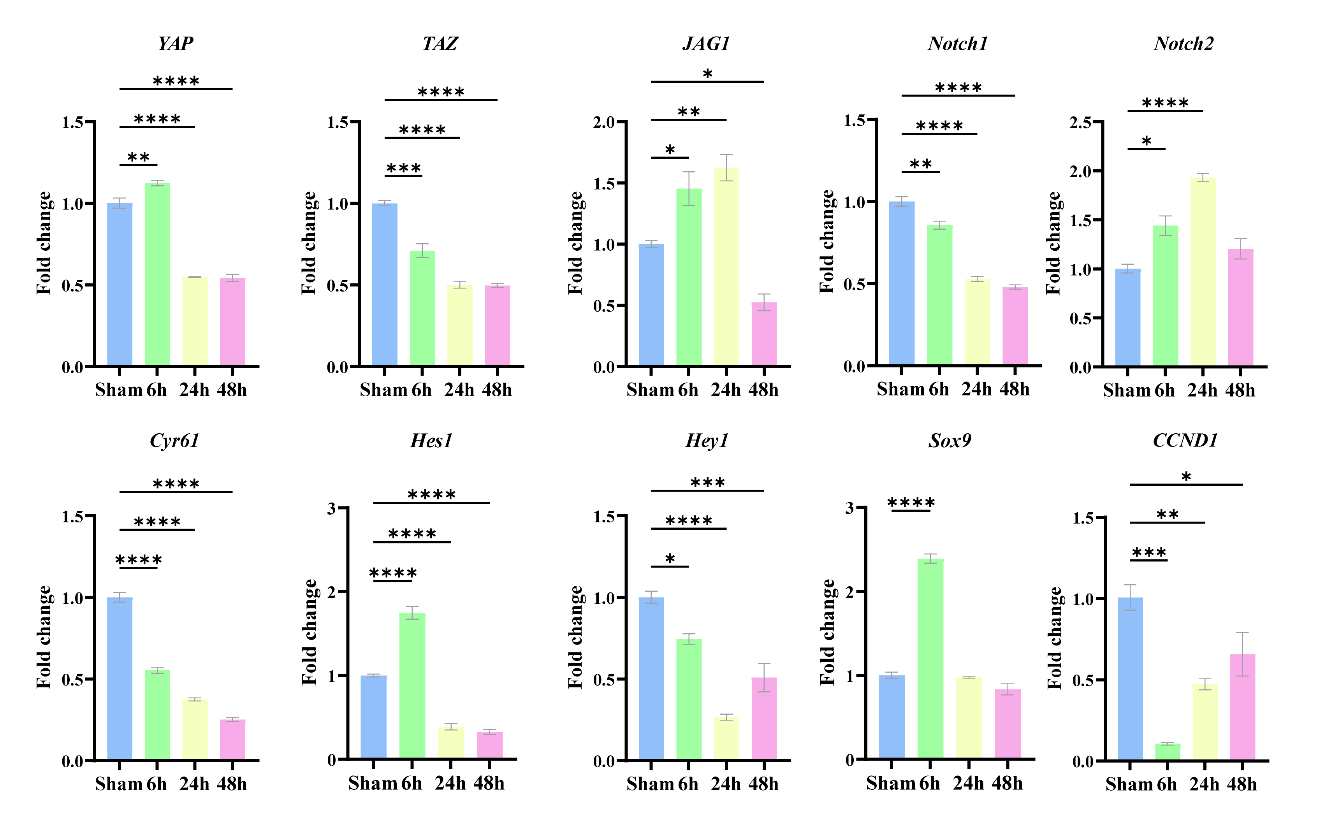
**2 Alterations in the genes of the YAP-Notch pathway in the hypertrophic side of liver tissue**

Sham, 6h, 24h, and 48h refer to the sampling time points from the hypertrophic side of liver tissue after surgery. Bar chart of YAP-Notch signaling pathway genes in hypertrophic liver tissue after surgery. Values are presented as the mean (X̅) ± standard error (SE), n=3.


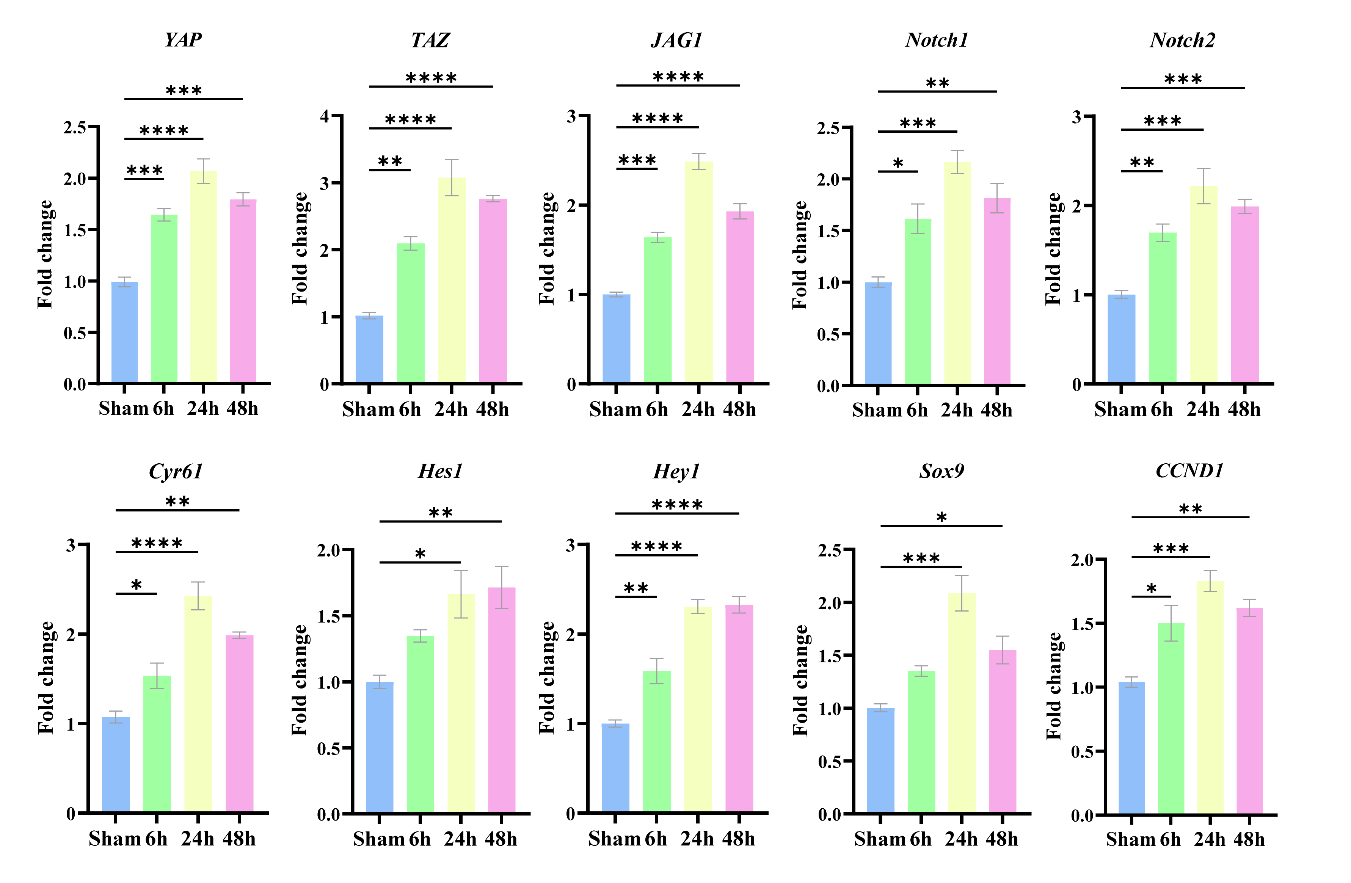
**Supplementary Fig.3 Alterations in the genes of the YAP-Notch pathway in the BRL-3A cell line (2 h) after exosomes treatment.**

Bar chart of YAP-Notch signaling pathway genes in the BRL-3A cell line (2 h) after exosomes treatment. Sham, 6h, 24h, and 48h refer to exosomes from the hypertrophic side of liver tissue after surgery. Values are presented as the mean (X̅) ± standard error (SE), n=3.
