# Supplementary figures and images for: Exosomes from liver progenitor cells carrying JAG1 activate notch signaling to promote liver regeneration in PVL rats
Source: Cell Death Dis. 2025 Aug 12;16(1):609. doi: 10.1038/s41419-025-07925-1 (PMC12343779; doi:10.1038/s41419-025-07925-1)

Fig.1D

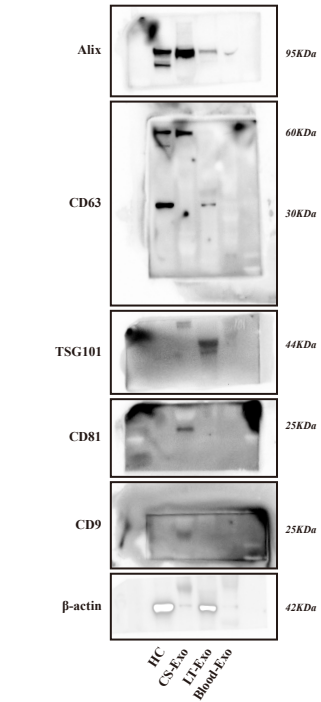

Fig.2F

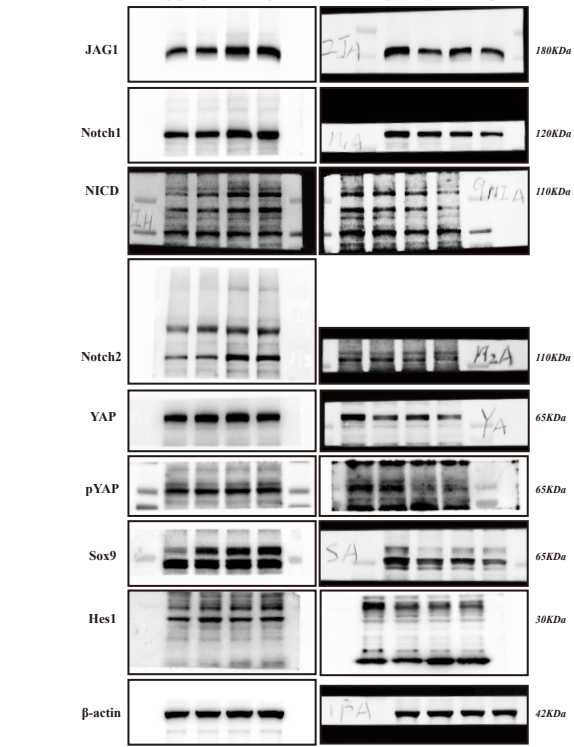

Fig.2G

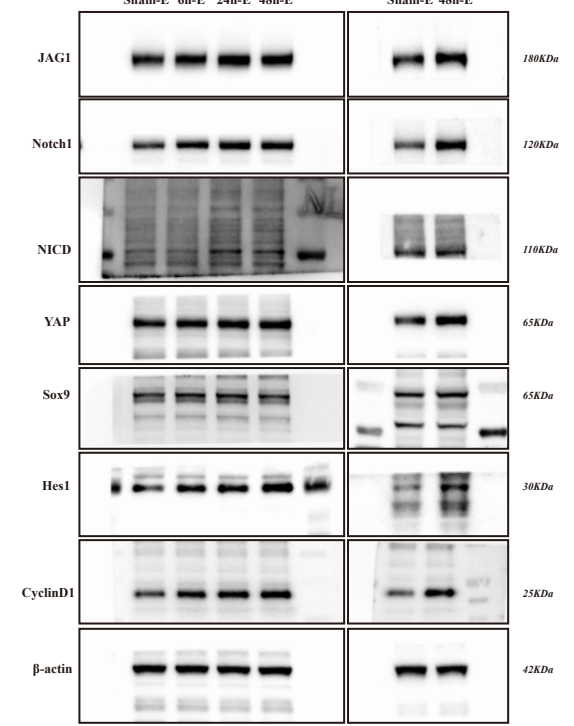

Fig.3A

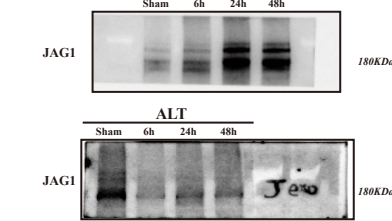

Fig.4C

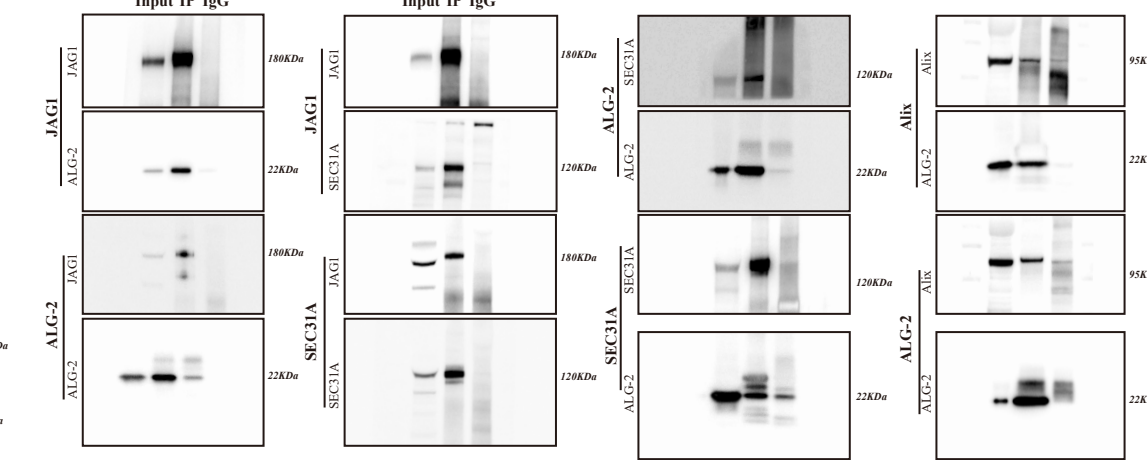

Fig.3J

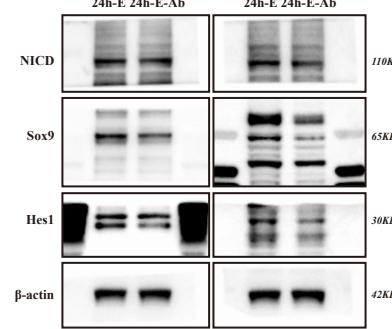

Fig.4E

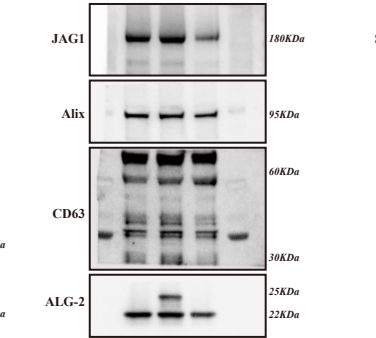

Fig.4F

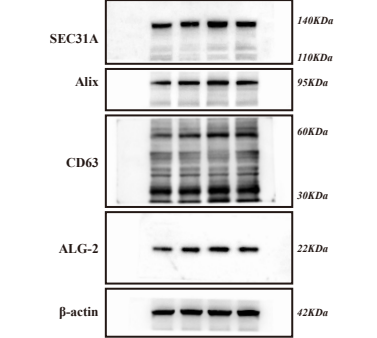

Fig.4G

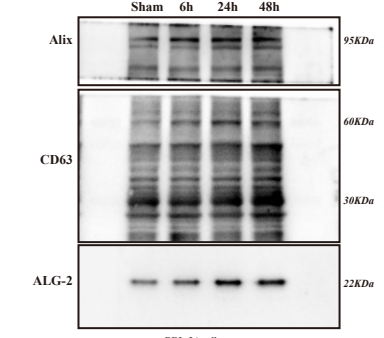

Fig.4D

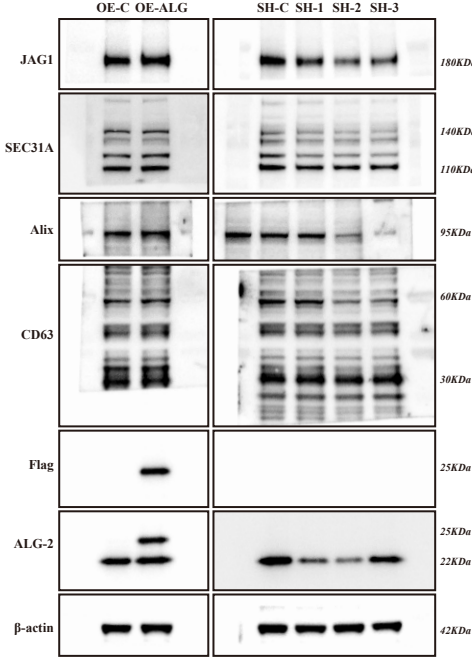

Fig.5E

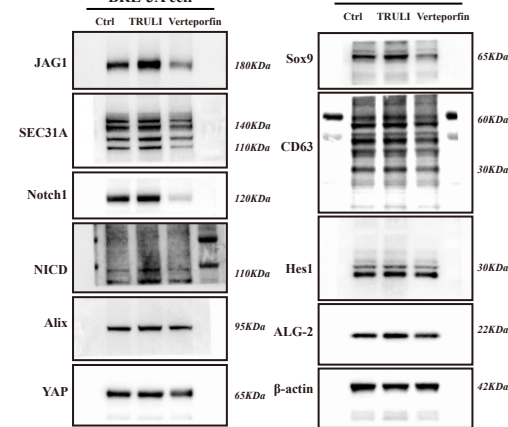

Fig.5F

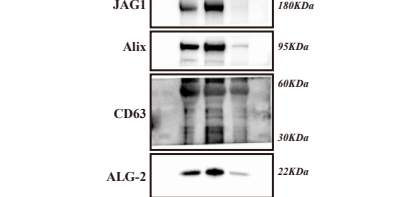

Fig.5K

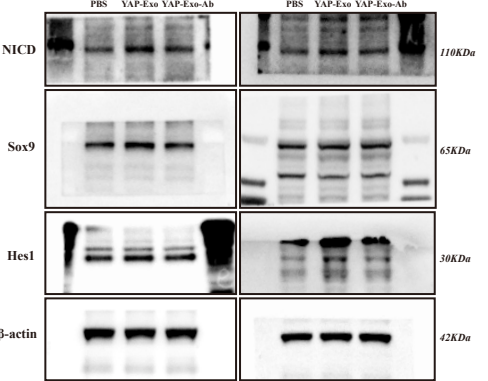

Supplement: Supplementary file 4 — Uncropped western blots [file 41419_2025_7925_MOESM4_ESM.pdf]
